# Drivers of tick community structure in a rhinoceros meta-population in Kenya

**DOI:** 10.1016/j.ijppaw.2026.101191

**Published:** 2026-01-14

**Authors:** Edward M. King'ori, Patrick I. Chiyo, Olgabeth N. Gitau, Fredrick Lala, Olivia Wesula Lwande

**Affiliations:** a Disease Diagnostics Laboratory, Veterinary and Capture Services Department, Kenya Wildlife Service, P.O. Box 40241, 00100, Nairobi, Kenya; b Wildlife Forensics and Genetics Laboratory, Veterinary and Capture Services Department, Kenya Wildlife Service, P.O. Box 40241, 00100, Nairobi, Kenya; c Kenyatta University, Department of Biochemistry, Microbiology and Biotechnology, P.O Box 43844, 00100, Nairobi, Kenya; d Wildlife Research and Training Institute (WRTI), P.O. Box 842, Naivasha, 20117, Kenya; e Department of Clinical Microbiology, Umeå University, 901 85, Umeå, Sweden; f Umeå Centre for Microbial Research, 901 87, Umeå, Sweden

**Keywords:** Tick-borne diseases, *Diceros bicornis*, *Ceratotherium simum*, Species-richness, Species-diversity, Hill-series

## Abstract

Understanding the structure and drivers of parasite communities including species assembly patterns, diversity, abundance, and aggregation is crucial in assessing the health of wild populations and the dynamics of host-parasite interactions within ecosystems. This study analyzed tick communities parasitizing the critically endangered black rhinoceros and the near threatened white rhinoceros metapopulation in twelve sanctuaries in Kenya. A total of 14,302 ticks from 20 tick species across four genera, *Dermacentor* (1 species), *Rhipicephalus* (8 species), *Amblyomma* (8 species) and *Hyalomma* (3 species) were sampled from 372 rhinoceroses. The most dominant species included *Amblyomma gemma* (23.28 %), *Amblyomma sparsum* (22.28 %) and *Rhipicephalus pulchellus* (18.94 %). Six tick communities were identified based on similarity in relative tick species composition. Mean NDVI and temperature were the major drivers of tick communities. Asymptotic Hill-Shannon and Hill-Simpson tick diversity metrics were 8.12 and 6.26 respectively for the Kenyan rhinoceros metapopulation. Species diversity varied between sanctuaries with Nairobi National Park (NNP) having the highest diversity (Hill-Shannon: 6.35, Hill-Simpson: 5.8) and Sera Rhinoceros Sanctuary (SER) the lowest diversity, (1.83, 1.69). The Intensive Protection Zone (IPZ) and Nairobi National Park had the greatest species richness (14 and 13 respectively), while Sera Rhinoceros Sanctuary had the lowest (2). Spatial heterogeneity in NDVI and species abundance were major drivers of species richness and Hill-Shannon species diversity. The number of ticks per rhinoceros was highly variable with a mean (SD) of 38.53 + 40.59 ticks per host, indicating strong tick aggregation among hosts. Significant positive interspecies correlations suggest a great role of host factors in tick infestation. Environmental factors, including temperature, NDVI, and rainfall, influenced tick abundance. Host-related factors, such as age, and sex, also played critical roles. This research improves our understanding of rhinoceros tick communities, diversity, and abundance patterns, with implications for tick control, tick-borne disease surveillance and rhino conservation in Kenya.

## Highlights

• Tick diversity was high for Kenyan rhinoceros.
• Diversity was influenced by spatial heterogeneity in NDVI.
• Tick abundance was influenced by temperature, NDVI and host factors.

## Graphical abstract

**Figure:**
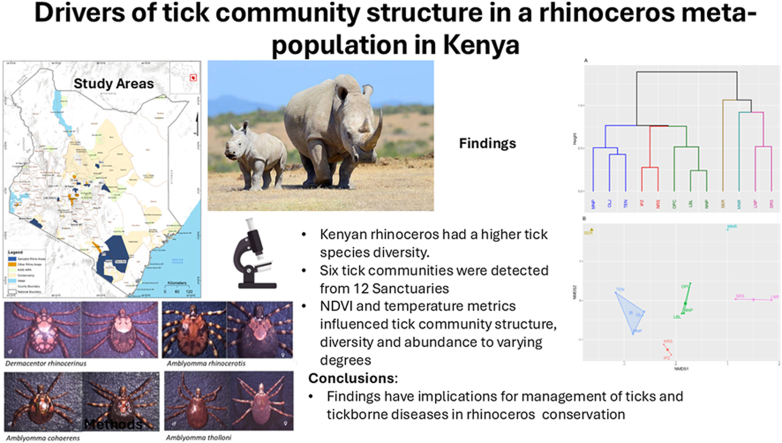

## 1 Introduction

Ticks are vectors of several important pathogens affecting terrestrial vertebrates and play important roles in the health, survival and reproductive success of mammalian (Fourie and Vrahimis, 1989; Hillegass et al., 2010; Patterson et al., 2013; Jones et al., 2019) and avian (Ramos et al., 2001; Hoodless et al., 2003; Boulinier and Danchin, 2008; Militão et al., 2024) host populations. Tick-borne diseases can cause significant economic losses to livestock (Eskezia and Desta, 2016) and can threaten wildlife populations (Cossu et al., 2024; Wiedeman et al., 2024). Ticks are also vectors of zoonotic diseases of concern to both human and animal health in Africa including Crimean Congo hemorrhagic fever (Goswami, 2014; Kuehnert et al., 2021) and Q fever (Vanderburg et al., 2014; Mangena et al., 2023). Tick abundance, diversity and distribution are vital metrics in predicting tick-borne disease epidemiology in respective hosts (Peralbo-Moreno et al., 2022), providing information on pathogen-specific vectors and spatial and temporal risk patterns of tick-borne infections.

Large mammals like rhinoceros can be sentinels for studies on ixodid tick diversity, distribution and abundance as their large body size provides a habitat for generalist and specialist tick species alike (McCoy et al., 2013; Esser et al., 2016; Horak et al., 2017; Merrill et al., 2018). Adult ixodid ticks are more common in large hosts as reproduction tends to occur in these hosts (van Wieren, 2016; van Wieren and Hofmeester, 2016; Sipari et al., 2024). Moreover, ticks may play important roles in large mammal conservation. For example, the African rhinoceros species (*Diceros bicornis* and *Ceratotherium simum*) suffer morbidity and mortality from tick-borne pathogens specifically *Babesia bicornis* and *Theileria bicornis,* when subjected to ecological and anthropogenic stressors (McCulloch and Achard, 1969; Nijhof et al., 2003) even though they are often asymptomatic carriers of these pathogens (Yam et al., 2018). African rhinoceros populations have fallen dramatically in the last several decades because of poaching. As a result, black rhinoceros are considered critically endangered while white rhinoceros are considered near threatened. Although efforts have focused on controlling poaching, rhino populations remain threatened (Love et al., 2017; Muturi et al., 2018; Dwyer et al., 2020), by diseases and parasitic infestations in intensely managed populations (Miller, 2017). Despite a few studies on the ixodid ticks infesting African rhinoceros from Southern Africa (Knapp et al., 1997; Heylen et al., 2013; Horak et al., 2017) no in-depth studies have focused on rhinoceros ticks from Eastern Africa or elsewhere.

Several environmental and host factors can influence the distribution, diversity, and bionomics of ticks and tick-borne pathogens. Among environmental factors, temperature, rainfall, humidity, Normalized Difference Vegetation Index (NDVI), and vegetation structure are considered important (Paul et al., 2016; Pascoe et al., 2019; McDonough and Holloway, 2020; Ma et al., 2024; Tian et al., 2024). Temperature and rainfall influence tick-development rate, hence their abundance. In Kenya, several tick species have discontinuous distribution that are largely thought to be driven by these environmental variables. The microclimates within the sanctuaries, influenced by vegetation type and density, can create suitable conditions for tick survival and reproduction (Perez et al., 2016). Additionally, the type of vegetation can impact the availability of host species, thereby influencing tick-host interactions and tick population dynamics (Frawley et al., 2024).

Tick abundance among individual hosts can display high variance, with tick burden higher in a few individual hosts and low or absent in others, a phenomenon referred to as aggregation (Gourbière et al., 2015). This is a common behavior for macro-parasites and is thought to be driven mostly by individual host traits such as species, age, sex, and health status. This phenomenon of aggregation can influence tick abundance and diversity. Host body size is positively correlated with tick abundance and species richness (Horak, 1997; Esser et al., 2016; Mysterud et al., 2021). African rhinoceros, being large and long-lived mammals, can provide stable and substantial blood meals for ticks, which is crucial for the ticks' life cycle. The movement and behavior of the different rhinoceros species, including their habitat preferences and social structures, can influence how ticks are distributed between species in the same landscape. The black and white rhinos differ in size and social structure, and in Kenya, they sometimes occur in the same sanctuary, making them an ideal system to test the influence of body size and social structure on tick diversity and abundance. Effective conservation and management strategies must consider these factors to maintain healthy rhinoceros populations and reduce the risk of tick-borne pathogen transmission.

The primary objective was to determine the species diversity and the distribution of hard ticks that parasitize black and white rhinoceros in twelve Kenyan rhinoceros sanctuaries. The specific objectives were to: 1). determine the diversity of ticks infesting rhinoceros in selected sanctuaries, 2). investigate patterns of tick aggregation and assess how competition or individual host characteristics might influence these patterns, 3). examine variation in tick species assemblages in different rhinoceros sanctuaries and 4). assess the influence of climate and vegetation on the abundance and diversity of tick species infesting rhinoceros. An understanding of the tick species that infest rhinos is necessary in the development of target-specific health management strategies that can help to ensure the continued survival of these iconic species.

## 2 Materials and methods

### 2.1 Ethics statement

This research was approved by the Ethics Committee of the Kenya Wildlife Service (KWS/BRM/5001), the authority responsible for wildlife protection and conservation in Kenya. All data were gathered during rhinoceros immobilization for management activities such as ear notching, translocation, and clinical treatment of injuries (due to snares and fights), as well as infections. Immobilization and translocation were carried out by veterinarians from the Kenya Wildlife Service (KWS), following the KWS protocol for rhinoceros immobilization and translocation, Wildlife Veterinary Practice guidelines of 2018, and the Veterinary Surgeons and Veterinary Para-professionals Act Cap 366 of the Laws of Kenya, which governs veterinary practice in Kenya.

### 2.2 Study populations and their locations

**Fig. 1:**
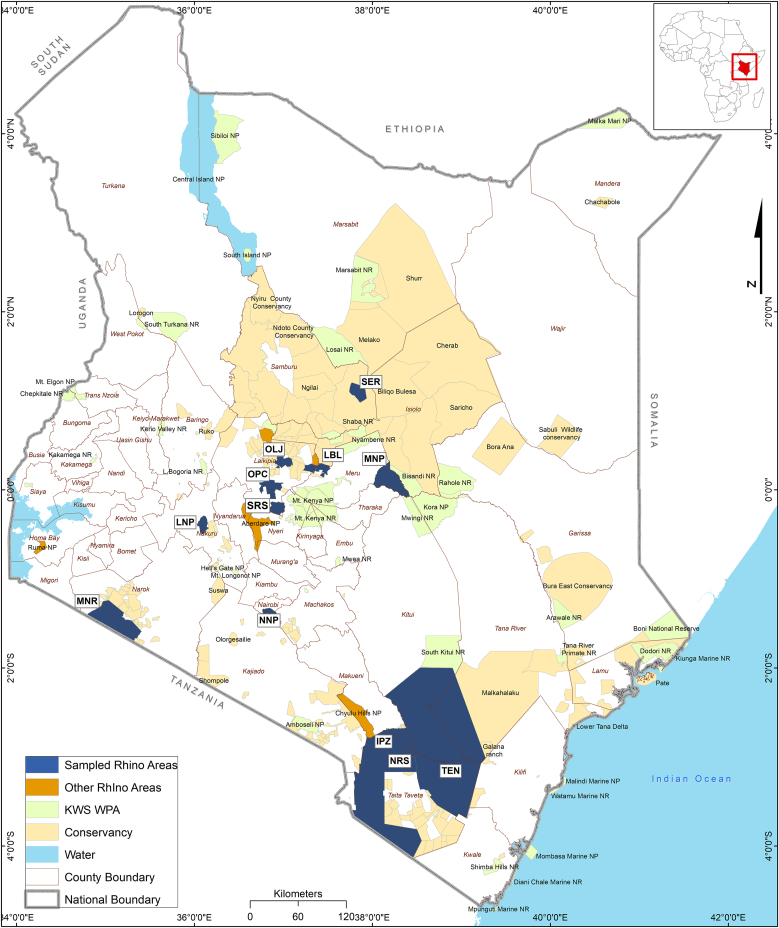
Location of the twelve rhinoceros sanctuaries sampled for tick diversity. OLJ is Ol Jogi, OPC is Ol Pejeta, LNP is Lake Nakuru National Park, SRS is Solio Rhinoceros Sanctuary, NNP is Nairobi National Park, MNR is Maasai Mara National Reserve, MNP is Meru National Park Rhinoceros Sanctuary, LBL is Lewa-Borana Landscape, SER is Sera Conservancy, IPZ is the Intensive Protection Zone in Tsavo West Natonal Park, NRS is Ngulia Rhinoceros Sanctuary in Tsavo West National Park and TEN is Tsavo East or Tsavo East National Park.

Both black and white rhinoceros were sampled from 12 sanctuaries in Kenya (Fig. 1). These populations were chosen from a total of 17 existing rhinoceros sanctuaries because these are among the sanctuaries with large rhinoceros populations with management activities that provided opportunities for immobilization and tick sampling (Fig. 1). These populations include Meru National Park (MNP), Nairobi National Park (NNP), Lake Nakuru National Park (LNP), Solio rhinoceros sanctuary (SRS), Ol Pejeta Conservancy (OPC), Ol Jogi Conservancy (OLJ), Lewa-Borana Landscape (LBL), Sera Wildlife Conservancy (SER), Maasai Mara National Reserve (MNR), Intensive Protection Zone in Tsavo West National Park (IPZ), Ngulia Rhino Sanctuary, Tsavo West National Park (NRS) and Tsavo East National Park (TEN).

The area, climate, vegetation, altitude, presence of a perimeter electric fence and access or proximity to livestock and rhinoceros number are provided in Table 1.

**Table 1:** Characteristics of the twelve Rhinoceros sanctuaries examined in this study.

Site	Area in km^2^ (Sanctuary)	Rainfall (mm)	Rhino Species	Fence	Livestock Access	Vegetation
IPZ	3000	200–700	37 black rhinoceros	None	No	Dense *Acacia-Commiphora* bushland and thorny thickets with interspersed open grasslands, and riverine forests of Doum palms
LBL	376.36	550	141 black and 123 white rhinoceros	Perimeter	Yes (integrated)	*Stipa dregeana* forest, *Acacia-Commiphora* woodlands, open grasslands
LNP	188	850	30 black and 130 white rhinoceros	Perimeter	No	Open grassland *Acacia* woodlands, *Tarchonanthus* bushland, deciduous & *Euphorbia* forests, riverine bushland
MNP	870 (48)	635–762	40 black and 79 white rhinoceros	Perimeter	No	Tall-grass savannah interspersed with Acacia woodlands, and extensive riverine forests
MNR	1821	650–1300	55 black rhinoceros	None	Yes	Grasslands with scattered Acacia woodlands, interspersed with dense riverine forests
NNP	117	800	97 black and 38 white rhinoceros	Partial perimeter	No	Deciduous forest, riverine thorn forests, shrubs, grasslands
NRS	90	600	143 black rhinoceros	Perimeter	No	Mixed bushland thickets, grasslands, shrubs, low tree, herbs
OLJ	235	460	64 black and 36 white rhinoceros	Perimeter	Yes (integrated)	Grassland, *Acacia* woodland, shrubs
OPC	93	850	166 black and 44 white rhinoceros	Perimeter	Yes (integrated)	Grassland, *Acacia* woodland, *Euclea* shrub, riverine woodland
SER	3500 (107)	355	22 black and 4 white rhinoceros	Perimeter	No	Bush and grasslands, *Acacia, Commiphora, Cordia* spp.
SRS	198.3 (76.9)	700	75 black and 500 white rhinoceros	Perimeter	Yes (integrated)	Grasslands savannahs interspersed with Acacia woodlands
TEN	13,747 (3300)	250–450	22 black rhinoceros	None	No	Grassland, bushland savannah, Acacia–Commiphora woodlands, *Premna*, *Bauhinia*, *Sericocomorpsis* scrub, *Delonix elata*, *Melia volkensii*

### 2.3 Tick sampling and identification

Ticks were obtained during rhinoceros management interventions, including translocation, ear notching, and clinical interventions (Table S1). During these activities, the rhinos were chemically immobilized, and samples were collected opportunistically. Rhinoceroses were immobilized using a Dan-Inject system equipped with 3 ml darts and 2.2 × 60 mm plain needles, to deliver anesthetic agents containing a combination of Etorphine HCl (0.98 %) (Captivon®) and Azaperone HCl (100 mg/ml). The drug dosage varied by age: animals between 1.5 and 2 years old received a combination of 1.5 mg of Etorphine and 50 mg of Azaperone, animals 2–3 years received 2.5 mg of Etorphine and 60 mg of Azaperone, animals 3–4 years were given 3.0 mg of Etorphine and 60 mg of Azaperone and animals older than 4 years or size were given 4 mg of Etorphine and 80 mg of Azaperone.

Once an animal is immobilized the capture team move in very quickly to ensure the animal is in sternal or lateral recumbency and blindfolded and ear plugs are inserted into the ear opening to minimize visual and auditory stimulation respectively. The capture team monitors vital parameters including respiration rate, heart rate, blood oxygen saturation and capillary refill rate by use of a pulse oximeter, while body temperature was monitored using s digital thermometer inserted into the rectum.

Three laboratory personnel per animal skillfully picked ticks for the period the animal was under anesthesia. This period was determined by the nature of intervention, for example, during rhino translocation, the period the animal was down varied between 10 and 20 min, while during ear notching the period was between 10 min and 15 min. The duration of anesthesia for the rhinoceros during clinical interventions depended on the type of procedures or injuries involved.

Once the purpose of immobilization is achieved (e.g. ear notching, lifted onto a crate for translocation or clinical treatment of wounds or infections), rhinoceros were administered with intravenous injections consisting of Butorphanol 10 mg and Naltrexone 75 mg for animals 1.5–3 years and 10 mg of Butorphanol and 100 mg of Naltrexone for animals over 3 years of age.

Ticks were collected from identified predilection sites, which included nostrils, ears, inside the vagina, prepuce and anus, below the tail and on the udder. Ticks were picked using a pair of forceps and put in universal bottles or cryovials. Ticks stored in universal bottles were preserved in 70 % ethanol, while those in cryovials were preserved in liquid nitrogen. In the laboratory, morphological identification of ticks was done using a stereo microscope to identify the species, sex and developmental stage. Standard morphological identifications keys were used to identify the tick species (Walker, 1974; Walker and Olwage, 1987; Walker et al., 2000; Horak et al., 2018).

### 2.4 NDVI, precipitation, and temperature

#### 2.4.1 Precipitation data

Precipitation data was sourced from the Copernicus Climate Change service using Google Earth Engine (GEE) (Gorelick et al., 2017; Muñoz-Sabater, 2019). Rainfall estimates, calculated over a grid with a spatial resolution of 11,132 square meters, include accumulated liquid and frozen water that lands on Earth's surface. Daily precipitation totals were extracted for each study site's central location using coordinates obtained via ArcMap 10.4's "polygon to point" tool (ESRI).

#### 2.4.2 Temperature data

Temperature data was derived from Copernicus Climate Change Service, Climate Data store (Muñoz-Sabater, 2019), accessed through GEE (Gorelick et al., 2017). Daily data on air temperature at 2 m above the surface of land was extracted for the central location of each study site at a spatial resolution of 11,132 square meters. Data on the minimum, mean and maximum temperatures in a day were recorded.

#### 2.4.3 NDVI data

Normalised Difference Vegetation Index (NDVI) data was sourced from the MODIS Terra Vegetation Product 16-day global dataset, which has a spatial resolution of 250 m (Didan, 2015) on GEE (Gorelick et al., 2017). This dataset is derived from the National Oceanic and Atmospheric Administration-Advanced Very High-Resolution Radiometer (NOAA-AVHRR) and calculated using atmospherically corrected bidirectional surface reflectance that have been masked for water, clouds, heavy aerosols, and cloud shadows. The downloaded product was processed in ArcGIS 10.4 (ESRI) using the "Extract Multi Values to Points" tool to obtain average monthly NDVI values for 30 random points at each study site.

### 2.5 Statistical analysis

#### 2.5.1 Environmental and management metrics

Daily records of precipitation in the form of rainfall were summed into monthly totals for each rhinoceros sanctuary and used as a predictor for tick species richness and species abundance at the individual animal level. For Normalized Difference Vegetation Index (NDVI), values were summed across thirty spatial points for each sanctuary to derive a monthly average of NDVI for each location. The Standard Deviation of the NDVI for each rhinoceros sanctuary was calculated and used as a measure of vegetation heterogeneity across the landscape in each sanctuary. Average temperature minimum, mean and maximum temperature were determined for each month and location and labelled as monthly minimum, monthly mean and monthly maximum temperature respectively. The mean and Standard Deviation of NDVI referred to as mean monthly NDVI and vegetation heterogeneity respectively were used alongside with monthly average temperature metrics and monthly total precipitation as independent variables in modeling tick species richness and abundance at the individual animal level. The rainfall totals for each month were summed up per calendar year for each location, and the mean annual rainfall was obtained for each location for an eight-year period from January 2017 to December 2024. Similarly, monthly mean NDVI, and monthly Standard Deviation across spatial points and monthly temperature metrics were also averaged over the same period. The resulting data was used as independent variables and species assemblages as a dependent variable in Redundancy Analysis (RDA) and mantel correlation analyses.

Data on rhinoceros numbers and density estimates were determined from rhinoceros population estimated from the Kenya National Wildlife census 2021 and rhinoceros areas available at the Kenya Wildlife Service (Table 1).

#### 2.5.2 Identification of tick communities and their environmental drivers

To identify similarities in tick communities or species assemblages from the different sanctuaries, we used Non-metric Multidimensional Scaling (NMDS), a statistical technique used for visualizing complex, high-dimensional data by reducing it to a lower-dimensional space while preserving the rank order of dissimilarities (Souza, 2025). The data transformation from a high-dimensional to a lower-dimensional space is achieved through an iterative process that minimizes a stress function, a measure of the divergence between the original dissimilarities and the distances in the reduced space. NMDS begins with a distance or dissimilarity matrix that quantifies the differences between pairs of observations. The NMDS algorithm iteratively adjusts the positions of points in a lower-dimensional space to minimize a stress function, which quantifies the discrepancy between the observed dissimilarities and the distances in reduced space. As a rule of thumb, lower stress value indicates a better fit. The NMDS scores were used to select the optimal number of clusters with similar ticks-assemblages using the Silhouette method in K-means clustering analysis to find the optimal number of clusters (Kassambara, 2017). In our analysis, relative species abundances within each sanctuary were used to estimate a Bray-Curtis dissimilarity measure that was subjected to NMDS ordination using the metaMDS function in the Vegan package of the R software for statistical computing (Oksanen et al., 2025).

Permutational Multivariate Analysis of Variance or PERMANOVA, a non-parametric technique was then employed to formally test if these observed groups are statistically significant, providing an associated p-value and R^2^ value (indicating the proportion of variance explained by the grouping factor) (Anderson, 2017). The vegan package in R was used for performing both of these analyses (Oksanen et al., 2025).

Bivariate mantel tests and Permutational Multivariate Analysis of Variance or PERMANOVA using the mantel and Adonis2 functions respectively were used to explore the influence of environmental variables on the difference in tick species composition across the 12 rhinoceros sanctuaries examined. Redundancy Analysis (RDA) was used to further confirm the influence of environmental variables on variation in tick species composition observed across sanctuaries (Capblancq and Forester, 2021). The environmental variables used included mean monthly NDVI, spatial variation in NDVI, Mean Annual rainfall, and mean temperature (monthly minimum, mean and maximum temperatures) averaged over eight years (January 2017 to December 2024) and management variables such as rhinoceros densities, whether sanctuary is fenced or not and presence of livestock. The RDA analyses were conducted using the “rda” and “Adonis” functions in the vegan package of the R software for statistical computing. Temperature metrics and rainfall were collinear and caused variance inflation, but the best subsets were selected using stepwise model selection using the ordistep function in vegan. To remedy multicollinearity, we run a Principal Component Analysis (PCA) on the set of collinear environmental variables and then used the resulting uncorrelated principal components as explanatory variables along with mean NDVI in the RDA analyses.

#### 2.5.3 Species diversity metrics

Empirical and asymptotic estimates of Hill-diversity metrics (Species richness, Hill–Shannon diversity and Hill–Simpson diversity) were calculated using the iNEXT Package of the R Statistical software (Hsieh et al., 2016). Hill diversity is a generalized weighted mean, or Hölder mean computed from the relative abundances of species in a sample (Roswell et al., 2021). Species richness uses an arithmetic rarity scale, which gives high leverage to, rare species; Hill–Simpson diversity uses a reciprocal scale, which shifts leverage to common species; Hill–Shannon uses a logarithmic scale and falls between the two.

We tested whether there were any statistically significant differences in the number Hill diversity indices (Species diversity, Hill-Shannon and Hill-Simpson) across sanctuaries using the simboot package in R (Scherer and Pallmann, 2024). Additionally, for sanctuaries maintaining both black and white rhinoceros, we tested the differences in species richness, and species diversity for the Hill-Shannon and Hill-Simpson diversity indices.

Linear regression analyses were used to test the influence of environmental and management factors on tick diversity metrics. Tick diversity metrics determined for each sanctuary such as species richness, Hill-Shannon and Hill-Simpson diversity indices were incorporated into the regression models as dependent variables whereas mean annual NDVI, average spatial heterogeneity in NDVI, average minimum, mean and maximum Temperature, rhinoceroses’ density, presence and absence of electric fencing, and presence of livestock in the sanctuaries were incorporated as independent variables. For species diversity we used the log transformed species count as a dependent variable. To test the relative influence of the independent variables, in a linear regression framework, a permutation of all possible covariate combinations was undertaken and the best model was selected based on AIC (Akaike Information Criterion), using MuMIn R package (Bartoń, 2023). The best model was selected from the subsets with the smallest AIC.

We also used the Generalized Linear Mixed Models (GLMM) approach to test the influence of environment and management variables on the species’ richness at the individual host level. We used a log link function assuming Poisson distributed error structure were conducted. The tick species richness per individual host was incorporated into the model as a dependent variable and sanctuary was incorporated as a random effect. As independent or explanatory variables, environmental factors such as ambient mean monthly temperature, monthly NDVI and monthly spatial heterogeneity in NDVI were employed along with host factors such as age and sex and tick abundance. To test the relative influence of the independent variables, GLMM were performed on a permutation of all possible covariate combinations and the best model was selected based on AIC using MuMIn R package (Bartoń, 2023).

#### 2.5.4 Patterns of tick aggregation

Data on the number of ticks per individual or the aggregate of all tick species per individual rhinoceros in each population was fitted into a negative binomial with a fitted mean and K parameter using the “fitdistrplus” package in R (Delignette-Muller and Dutang, 2015). The fit of the data to the estimated negative binomial distribution was tested using the Chi-square statistics in the “fitdistrplus” package in R (Delignette-Muller and Dutang, 2015) and using an R-based interactive web service, the Quantitative Parasitology on the Web (QPweb) (Reiczigel et al., 2019). The negative binomial distribution was used to model the aggregation of ticks, with the dispersion parameter k estimated and used as an index of aggregation (Shaw et al., 1998). A smaller k value signifies greater aggregation, with many hosts having few parasites while a few hosts carry many parasites (Shaw et al., 1998). This distribution is particularly useful for analyzing highly aggregated data. Additionally, the variance to mean ratio was calculated to further quantify the degree of aggregation. Higher ratios indicate that the data are more aggregated, providing another layer of analysis for understanding parasite distributions.

To complement these measures, Poulin's discrepancy index was employed to assess the heterogeneity of parasite distribution among hosts. This index evaluates the discrepancy between observed and expected distributions under a given null model, such as Poisson or binomial distributions. A higher discrepancy index indicates greater aggregation, revealing the extent to which parasite loads deviate from randomness. Together, these statistical tools provide a comprehensive picture of parasite aggregation, enhancing our understanding of host-parasite dynamics.

Spearman's rank correlation was utilized to quantify these relationships. Positive and negative correlations in the abundance of different tick species across individuals were related to factors facilitating tick aggregation. Positive correlations suggest that individuals heavily infested with one tick species are likely to host high numbers of another species, highlighting shared environmental factors or host characteristics that favor increased tick burdens. Conversely, negative correlations imply competitive exclusion, where the presence of one tick species reduces the abundance of another. This may result from limited resources, direct competition, or differing microhabitat preferences on the host.

#### 2.5.5 Tick prevalence, abundance and factors influencing abundance

Prevalence patterns of tick species infestation among rhinoceros species and sanctuary were determined as the proportion (expressed as a percentage) of all observed rhinoceros infested with a specific tick species or all tick species combined using the traditional Clopper-Pearson method was used to determine the Confidence interval for prevalence (Reiczigel et al., 2019). First, we assessed whether the prevalence of tick species was different in black and white rhinoceros species using a chi-square test of association. Sanctuary and tick species were incorporated into the models as random effects. We used data for rhinoceros from sanctuaries containing both black and white rhinoceros in sympatry. Once we confirmed that rhinoceros species had no influence on tick prevalence, we analyzed tick prevalence for all rhinoceros irrespective of species.

To assess the influence of environmental and hosts related factors on tick abundance, Generalized Linear Mixed Models (GLMM) with a log link function assuming Poisson distributed error structure were conducted. The abundance of different tick species or all species combined were incorporated into models as dependent variables and Sanctuary was incorporated as a random effect. As independent explanatory variables, environmental factors such as ambient mean monthly temperature, monthly NDVI and monthly spatial heterogeneity in NDVI were employed along with host factors such as species of rhinoceros, age and sex. To test the relative influence of the independent variables, GLMM were performed on a permutation of all possible covariate combinations and the best model was selected based on AIC using MuMIn R package (Bartoń, 2023). The best model was selected from the subsets with the smallest AIC. There was sufficient data for 10 of the 20 tick species for these analyses.

## 3 Results

### 3.1 Tick species composition: communities, patterns and drivers among sanctuaries

**Fig. 2:**
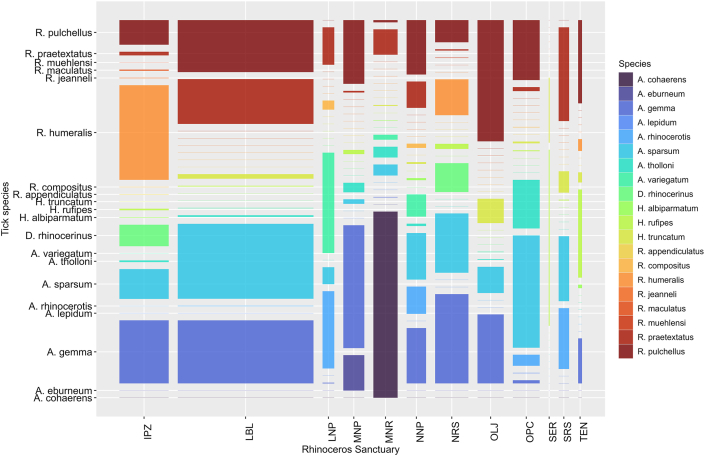
Mosaic plot showing the distribution and species richness of tick species across twelve rhinoceros Sanctuaries in Kenya.

About 14,302 ticks from 372 rhinoceros (239 black and 133 white rhinoceros) in 12 rhinoceros sanctuaries were sampled and identified. These ticks comprised 20 tick species (Fig. 2) from four genera: *Dermacentor* (1 species), *Rhipicephalus* (8 species), *Amblyomma* (8 species) and *Hyalomma* (3 species). Five tick species made up 81.41 % of the tick burden on rhinoceros, and these included *Amblyomma gemma* (23.28 %), *Amblyomma sparsum* (22.28 %), *Rhipicephalus pulchellus* (18.94 %), *Rhipicephalus praetextatus* (10.14 %), and *Rhipicephalus humeralis* (6.77 %).

**Fig. 3:**
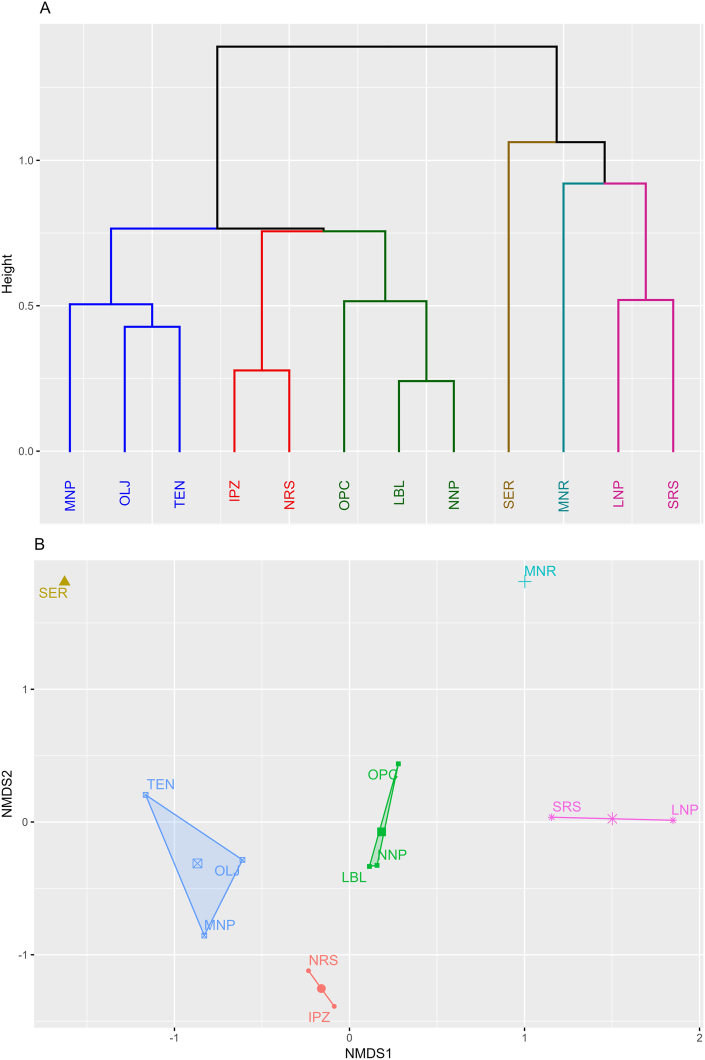
Hierarchical clustering dendrograms and NMDS ordination of the similarity in tick species composition across 12 rhinoceros Sanctuaries. Dendrogram (A) and NMDS (B) plots are shown for relative species counts and Bray-Curtis's distance matrix. Clustering of Sanctuaries is based on Ward's minimum variance method using K-means clustering.

NDMS dimension reduction with a stress factor and 0.10684 in two-dimensional space and silhouette clustering algorithm using K-clustering method revealed six tick species assemblages (Fig. 3): 1). OPC, LBL and NNP, 2). OLJ, MNP and TEN, 3). LNP and SRS, 4). IPZ and NRS and Singletons 5). SER and 6). MNR (Fig. 3). These groupings explained 82.05 % of variation in relative abundance in tick species composition across rhinoceros sanctuaries in Kenya (F_5,6_ = 5.484, p < 0.001). Dominant ticks varied by sanctuary reflecting groupings in tick assemblages. *A. sparsum* and *R. pulchellus* dominated group one: OPC had *A*. *sparsum* (46.55 %), *R*. *pulchellus* (23.83 %) and *Amblyomma tholloni* (20.11 %), while LBL had *A*. *sparsum* (31.05 %) *A*. *gemma* (26.11), *R*. *pulchellus* (21.52 %) and *R*. *praetextatus* (18.53). NNP had three codominant ticks: *A*. *gemma* (22.91 %), *R*. *pulchellus* (22.51 %), and *A*. *sparsum* (19.24 %). Group 2 tick-assemblage was dominated by *A. gemma* and *R. pulchellus*. MNP had *A*. *gemma* (50.97 %), *R*. *pulchellus* (26.39 %) and *Amblyomma eburneum* (14.65 %) as the most abundant ticks. OLJ had *R*. *pulchellus* (50.68 %) and *A*. *gemma* (28.02 %) as the most common ticks. Rhinoceros from TEN had *Hyalomma rufipes* (36.55 %) and *R*. *pulchellus* (34.48 %) and *A*. *gemma* (18.6 %) as the dominant ticks. Group 3 rhinoceros sanctuaries (LNP and SRS) were dominated by *Amblyomma rhinocerotis* and *R. praetextatus*. LNP was dominated by *Amblyomma variegatum* (41.61 %), *A*. *rhinocerotis* (32.03 %) and *R*. *praetextatus* (15.47 %). SRS was dominated by *R*. *praetextatus* (36.69 %), *A*. *sparsum* (28. 42 %) and *A*. *rhinocerotis* (25.58 %). The fourth group of sanctuaries (IPZ and NRS) were dominated *A. gemma*, *A. humeralis* and *A. sparsum*. IPZ had *R*. *humeralis* (39.24 %), *A*. *gemma* (26.13 %) and *A*. *sparsum* (12.30 %), while NRS was dominated by *A*. *gemma* (37.01 %), *A*. *sparsum* (24.54 %), and *R*. *humeralis* (14.82 %) as the most common ticks. *Dermacenter rhinocerinus* was found also found at IPZ, and NRS only.

MNR formed its own cluster dominated by *Amblyomma cohaerens* (77.28 %) which was not found elsewhere among the rhinoceros sanctuaries examined. *Rhipicephalus praetextatus* (10.47 %) was somewhat common. SER another sanctuary forming its own tick assemblage was dominated by *Hyalomma rufipes* (73.08 %) and *Hyalomma truncatum* (26.92 %). SER was also unique in that *A*. *gemma* and *A. sparsum* found in nearly all sanctuaries was absent in SER.

Climatic and vegetation variables examined across the 12 sanctuaries in this study suggest these locations have a mostly semi-arid to arid climate with generally cooler temperatures, and variable rainfall patterns for the period January 2017 to December 2024 (Table 2). These variables along with other variables like, presence of an electric fence, presence or proximity to livestock were used as independent variables models of tick community structure.

**Table 2:** Remotely sensed vegetation and climatic variables average by Rhinoceros Sanctuary (January 2017–December 2024).

Sanctuary	Mean Total Annual Rainfall ± SD in mm	Mean temperature ± SD in Celsius	Minimum temperature ± SD in Celsius	Maximum Temperature ±SD in Celsius	Mean NDVI ± SD	Spatial SD mean ± SD
IPZ	642.95 ± 178.81	23.47 ± 0.4	18.72 ± 0.32	28.88 ± 0.60	0.399 ± 0.086	0.086 ± 0.042
LBL	1311.72 ± 470.54	17.55 ± 0.28	13.26 ± 0.29	22.35 ± 0.40	0.393 ± 0.059	0.059 ± 0.021
LNP	1129.67 ± 266.08	18.67 ± 0.39	13.76 ± 0.29	24.01 ± 0.58	0.579 ± 0.072	0.072 ± 0.019
MNP	628.55 ± 264.03	25.77 ± 0.35	21.06 ± 0.28	30.92 ± 0.43	0.468 ± 0.075	0.075 ± 0.028
MNR	745.44 ± 155.21	20.78 ± 1.51	16.55 ± 2.92	27.06 ± 1.78	0.545 ± 0.065	0.065 ± 0.02
NNP	638.06 ± 263.09	19.17 ± 0.36	14.83 ± 0.27	24.23 ± 0.54	0.452 ± 0.087	0.087 ± 0.022
NRS	579.14 ± 231.45	24.57 ± 0.37	20.1 ± 0.33	29.94 ± 0.54	0.432 ± 0.053	0.053 ± 0.033
OLJ	688.91 ± 295.7	18.93 ± 0.38	13.48 ± 0.33	24.62 ± 0.57	0.398 ± 0.051	0.051 ± 0.018
OPC	852.64 ± 384.81	17.64 ± 0.42	12.31 ± 0.33	23.42 ± 0.66	0.488 ± 0.076	0.076 ± 0.018
SER	307.71 ± 190.24	25.72 ± 1.11	21.91 ± 2.84	31.17 ± 1.26	0.283 ± 0.035	0.035 ± 0.027
SRS	1852.8 ± 453.17	16.47 ± 0.41	12.35 ± 0.37	21.29 ± 0.49	0.508 ± 0.082	0.082 ± 0.021
TEN	448.71 ± 155.54	25.76 ± 0.31	21.57 ± 0.29	31.28 ± 0.43	0.297 ± 0.062	0.062 ± 0.037

To perform PERMANOVA, the assumption of homogeneity of multivariate dispersions is required and was tested using the “betadisper” function in vegan. Indeed, the homogeneity of multivariate dispersions was confirmed (F_5,6_ = 2.481, R^2^ = 0.0212, P = 0.142). Bivariate analyses using Mantel correlations and PERMANOVA analyses revealed the effect of mean monthly NDVI, mean monthly minimum temperature, mean monthly mean temperature and mean monthly had a strong influence on differences in tick species assemblages found in a rhinoceros metapopulation in Kenya (Table 3). Rhinoceros density, presence of cattle in the sanctuary, and whether a sanctuary is fenced or not had no influence on differences in species assemblages between sanctuaries (Table 3). Redundancy Analysis, RDA following stepwise elimination of variables revealed that mean NDVI was the best variable in a model and explained 14.87 % of variation in tick species assemblages across sanctuaries (F_1,10_ = 2.922, R^2^ = 0.2261, R^2^adj = 0.148, p < 0.001). The temperature metrics and rainfall, although significant in bivariate analyses, were not selected because of variance inflation attributed to multicollinearity (Pearson r ≥ 0.7 among temperature metrics and rainfall).

**Table 3:** The influence of temperature, Normalized Difference Vegetation Index (NDVI), rainfall, rhinoceros density and presence of cattle on tick communities evaluated using bivariate Mantel and PERMOVA tests.

Predictor variables	Mantel Tests		PERMAVO Tests			
	Mantel r	P value	D.F	R^2^	F	Pr(>F)
Bivariate models						
Mean NDVI	0.63	**0.0001**	1, 10	0.254	3.399	**0.0013**
Spatial heterogeneity in NDVI	0.12	0.2527	1, 10	0.127	1.460	0.2001
Minimum Temperature	0.27	**0.0134**	1, 10	0.207	2.608	**0.0174**
Mean Temperature	0.24	**0.0244**	1, 10	0.216	2.752	**0.0099**
Maximum temperature	0.23	**0.0291**	1, 10	0.217	2.766	**0.0125**
Rainfall	0.23	0.1681	1, 10	0.202	2.525	**0.0246**
Rhinoceros density	0.07	0.3039	1, 10	0.082	0.896	0.4774
Cattle presence	0.01	0.3792	1, 10	0.106	1.183	0.3161
Electric fencing	0.09	0.3086	1, 10	0.074	0.801	0.5966

**Fig. 4:**
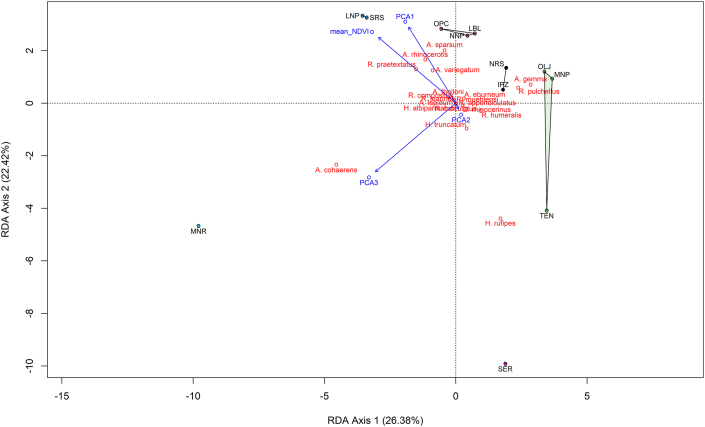
The redundancy analysis (RDA) graph illustrates how average NDVI and temperature metrics affect the composition of different tick species. Temperature metrics are represented by PCA1, PCA2, and PCA3. Tick species marked in red and rhinoceros sanctuaries are colored differently in terms of similarity in tick species composition.

A Principal Component Analysis (PCA) performed on the standardized minimum, mean and maximum temperatures revealed major unconstrained patterns of temperature variation among sites. The first principal component (PC1) explained 99.28 % of the total variance, while the second principal component (PC2) accounted for 0.62 %. PC1 had moderate and negative correlation with minimum (r = −0.576), maximum (r = −0.577) and mean temperature (r = −0.579) while PC2 had a strong positive correlation with minimum temperature (r = 0.730) and a negative correlation with maximum temperature (r = −0.682). PC3 contributed to 0.098 % of total variation and was negatively correlated with mean temperature (r = −0.814). These unconstrained PCA scores provided data for subsequent Redundancy Analysis, which explicitly tested the influence of the temperature metrics on the observed patterns of tick community composition. The redundancy analysis (RDA) revealed a significant influence of environmental variables on tick community composition (F _(4,7)_ = 2.915, P < 0.001). The three selected environmental variables (mean NDVI, mean temperature, minimum temperature and maximum temperature) explained 60.48 % of the total variance in the relative composition of tick species (Table 4). The biplot (Fig. 4) illustrates that *A. rhinocerotis*, *A. praetextatus* and *A. variegatum* were positively associated with mean NDVI, while *A. cohaerens* was correlated with PC3. Standardized canonical coefficients are provided in Table 4.

**Table 4:** A multivariate analysis of drivers of tick communities using model selection and PCA to address issues multicollinearity.

Predictor variables	Degrees of freedom	Percent variance explained	F statistic	Pr(>F)
*RDA analyses of variables positive in bivariate mantel tests*				

Mean temperature	1	18.17	3.39	0.0010
Maximum temperature	1	13.21	2.46	0.0260
Minimum temperature	1	17.73	3.31	0.0050
Mean NDVI	1	13.38	2.50	0.0060
Residual	7	37.51		

*Using PCA to address issues of multicollinearity with correlated temperature metrics*				

PCA1	1	18.23	3.40	0.0001
PCA2	1	7.03	1.31	0.2444
PCA3	1	23.85	4.45	0.0002
Mean NDVI	1	13.38	2.50	0.0076
Residual	7	37.51		

*Variable selected through backward and forward variable elimination*				

Mean NDVI	1	22.61	2.92	0.0001
Residual	10	77.39

### 3.2 Tick species diversity among sanctuaries, patterns and causes

The overall empirical and asymptotic estimates of tick species richness of Kenyan rhinoceros meta-population, were 20 and 24 species respectively. However, when these were calculated by rhinoceros Sanctuary, the empirical and asymptotic estimates of species richness were high for IPZ (13, 14), Nairobi National Park (10,10), OPC (10, 11) MNR (9,10), LBL (9, 9) and NRS (8, 8), moderate for MNP (7,7), LNP (6, 6) TEN (6,6), OLJ (6,6) and SRS (5,5). The species richness was lowest for Sera Wildlife Conservancy (2, 2) respectively (Table 5).

**Table 5:** Hill diversity measures of ticks infesting rhinoceros in Kenyan Sanctuaries.

	Sanctuary	Empirical Hill diversity parameter (95 % LCL─UCL)	Estimated Hill diversity parameter (95 % LCL─UCL)
**Species richness**	IPZ	13 (10 ─ 16)	14 (13 ─ 19)
	LBL	9 (9 ─ 9)	9 (9 ─ 9)
	LNP	6 (4 ─ 8)	6 (6 ─ 7)
	MNP	7 (7 ─ 7)	7 (7 ─ 7)
	MNR	9 (7 ─ 11)	10 (9 ─ 12)
	NNP	10 (10 ─ 10)	10 (10 ─ 10)
	NRS	8 (7 ─ 9)	8 (8 ─ 9)
	OLJ	6 (4 ─ 8)	6 (6 ─ 9)
	OPC	10 (8 ─ 12)	11 (10 ─ 13)
	SER	2 (2 ─ 2)	2 (2 ─ 2)
	SRS	5 (3 ─ 7)	5 (5 ─ 6)
	TEN	6 (5 ─ 7)	6 (6 ─ 7)
	All	20 (14 ─ 26)	24 (16 ─ 33)
**Hill-Shannon diversity**	IPZ	4.89 (4.71 ─ 5.08)	4.91 (4.74 ─ 5.09)
	LBL	4.41 (4.34 ─ 4.48)	4.41 (4.34 ─ 4.49)
	LNP	3.82 (1.89 ─ 5.74)	3.84 (3.61 ─ 4.07)
	MNP	3.58 (3.37 ─ 3.79)	3.59 (3.37 ─ 3.82)
	MNR	2.37 (2.20 ─ 2.55)	2.38 (2.22 ─ 2.54)
	NNP	6.55 (6.25 ─ 6.85)	6.59 (6.33 ─ 6.85)
	NRS	4.84 (4.66 ─ 5.02)	4.86 (4.69 ─ 5.02)
	OLJ	3.30 (3.16 ─ 3.44)	3.31 (3.19 ─ 3.43)
	OPC	3.85 (3.65 ─ 4.05)	3.87 (3.68 ─ 4.06)
	SER	1.79 (1.48 ─ 2.10)	1.83 (1.52 ─ 2.13)
	SRS	3.66 (2.04 ─ 5.27)	3.67 (3.5 ─ 3.85)
	TEN	4.00 (3.53 ─ 4.47)	4.07 (3.63 ─ 4.51)
	All	8.12 (8.00 ─ 8.24)	8.12 (8.00 ─ 8.25)
**Hill Simpson diversity**	IPZ	3.91 (3.74 ─ 4.08)	3.91 (3.75 ─ 4.08)
	LBL	4.07 (4.01 ─ 4.13)	4.07 (4.02 ─ 4.13)
	LNP	3.27 (1.37 ─ 5.17)	3.29 (3.05 ─ 3.52)
	MNP	2.83 (2.65 ─ 3.02)	2.84 (2.64 ─ 3.04)
	MNR	1.63 (1.53 ─ 1.73)	1.63 (1.54 ─ 1.73)
	NNP	5.76 (5.48 ─ 6.05)	5.80 (5.52 ─ 6.08)
	NRS	4.13 (3.92 ─ 4.34)	4.14 (3.94 ─ 4.34)
	OLJ	2.81 (2.65 ─ 2.96)	2.81 (2.67 ─ 2.95)
	OPC	3.11 (2.95 ─ 3.28)	3.12 (2.96 ─ 3.28)
	SER	1.65 (1.26 ─ 2.04)	1.69 (1.27 ─ 2.12)
	SRS	3.39 (1.83 ─ 4.96)	3.41 (3.20 ─ 3.63)
	TEN	3.43 (3.01 ─ 3.86)	3.49 (3.08 ─ 3.9)
	All	6.26 (6.15 ─ 6.36)	6.26 (6.15 ─ 6.37)

Lower Confidence Level (LCL)─Upper Confidence Level (UCL).

For the entire Kenyan rhinoceros meta-population, asymptotic Hill-Shannon and Hill-Simpson diversity metrics respectively were 8.12 and 6.26. The asymptotic Hill-Shannon and Hill-Simpson diversity metrics respectively were highest for Nairobi National Park (6.55, 5.76), IPZ (4.91, 3.91) and NRS (4.88, 4.14) and were lowest for Sera Conservancy (1.83, 1.69), Meru Rhinoceros Sanctuary (2.13, 1.53), and Maasai-Mara National Reserve (2.40, 1.63). Significant differences in species richness (Table S2), Hill-Shannon (Table S3) and Hill-Simpson (Table S3) diversity metrics were limited and restricted to differences between IPZ, NRS, and NNP from the rest of the sanctuaries.

In sympatric populations of black and white rhinoceros residing in MNP, NNP, LNP, OLJ, OPC and LBL, there was no statistical differences in asymptotic species richness (Black rhinoceros: 11, 95 % CI: 11.00–11.99; White rhinoceros 11, 95 % CI:11.00–11.90; P = 0.882), Hill-Shannon (Black rhinoceros 5.99, 95 % CI: 5.875–6.106; White rhinoceros 5.214, 95 % CI: 5.095–5.333, P = 0.677) and Hill-Simpson (Black rhinoceros 4.987, 95 % CI: 4.871–5.103; White rhinoceros 4.257, 95 % CI:4.163–4.352; P = 0.733) species diversities between white and black rhinoceros respectively.

**Fig. 5:**
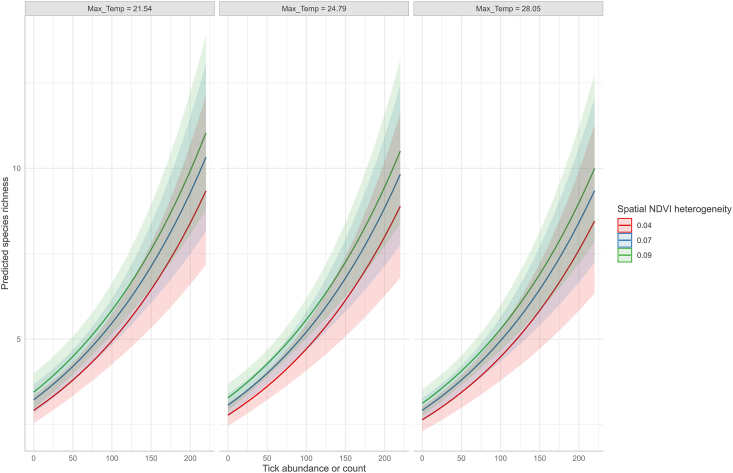
Marginal effects of mean monthly maximum temperature and tick abundance on predicted species richness.

Linear regression and model selection analyses indicated that variation in the log of species richness among sanctuaries was best accounted for by a model incorporating spatial heterogeneity in NDVI (F_1,10_ = 7.96, Adj R^2^ = 0.3875, P = 0.01812, Table 6). The log of species richness increased with NDVI heterogeneity, and the model explained 38.75 % of variation in log of observed species richness among rhinoceros sanctuaries. None of the other variables—such as annual rainfall, average monthly minimum temperature, average monthly temperature, average monthly maximum temperature, rhinoceros density, or the presence of cattle or fencing — showed statistical significance. A positive relationship was also found between observed Hill-Shannon tick diversity across sanctuaries and spatial heterogeneity in NDVI (F_1, 10_ = 5.522, R^2^_adj = 0.291, P = 0.041; see Table 6). This model explained 29.13 % of variation in observed Hill-Shannon tick diversity across sanctuaries. Spatial heterogeneity in NDVI showed a similar influence on observed Hill-Simpson diversity, but this effect was not statistically significant (F_1, 10_ = 3.793, Adj R^2^ = 0.2025, P = 0.080; see Table 6). At the individual rhinoceros level, accounting for sanctuary level effects in a GLMM framework, the influence of temperature metrics (average monthly minimum, mean and maximum temperatures), total monthly rainfall, Normalized Difference Vegetation Index (NDVI) metrics (monthly NDVI and spatial heterogeneity in NDVI), rhinoceros age, sex, and tick burden, on tick species richness was tested. Model selection results revealed the positive relationship between spatial heterogeneity in NDVI and the tick burden on species richness (Fig. 5). All the other factors were not significant. In a model where tick burden was not included, monthly NDVI and monthly spatial heterogeneity in NDVI were positively correlated with tick species richness while monthly maximum temperature negatively influenced tick species richness (Table 6).

**Table 6:** Best models showing coefficients of independent variables that best explain patterns of species richness, observed Hill-Shannon and Hill-Simpson diversity among Sanctuaries.

Variable	Estimate	Std. Error	t value	Pr(>|t|)
**Linear regression models for tick diversity metrics at the sanctuary level**				

Log *of Observed Species Richness*				
Intercept	1.939	0.1085	17.878	<0.0001
Spatial heterogeneity in NDVI	0.3196	0.1133	2.821	0.0181
*Observed Hill-Shannon Diversity*				
Intercept	3.921	0.298	13.18	0.0001
Spatial heterogeneity in NDVI	0.73	0.311	2.35	0.0406
*Observed Hill-Simpson Diversity*				
Intercept	3.333	0.288	11.569	0.0001
Spatial heterogeneity in NDVI	0.586	0.301	1.948	0.0801

**Species richness at the individual host level**				

*Excluding tick abundance*				
Intercept	1.188	0.071	16.761	0.000
Mean monthly NDVI	0.100	0.036	2.808	0.005
Heterogeneity in NDVI	0.135	0.038	3.583	0.000
Maximum temperature	−0.083	0.047	−1.756	0.079
*Including tick abundance into the model of covariates*				
Intercept	1.188	0.044	27.267	0.000
Heterogeneity in NDVI	0.087	0.032	2.720	0.007
Tick abundance	0.215	0.027	8.088	0.000
Maximum temperature	−0.050	0.035	−1.428	0.153

### 3.3 Patterns of rhinoceros infestation by ticks: aggregation, prevalence and abundance

**Fig. 6:**
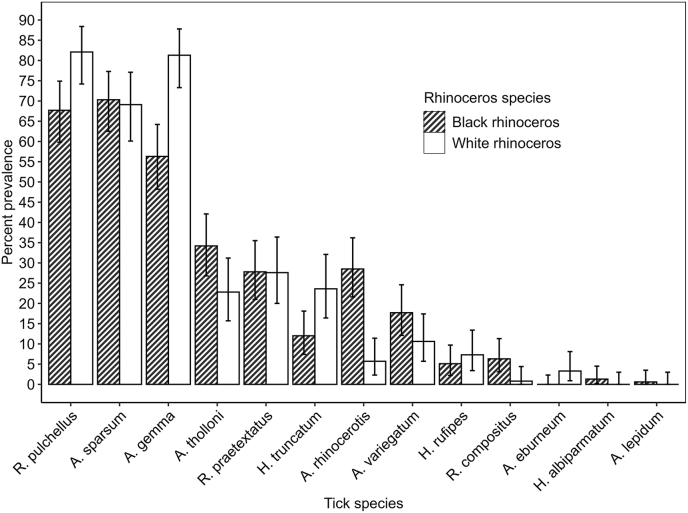
Variation in tick species prevalence and 95 % Confidence interval bars between sympatric white and black rhinoceros from six rhinoceros sanctuaries (LBL, LNP, MNP, NNP, OLJ, OPC).

All rhinoceros examined in the metapopulations were tick infested with a prevalence of 100 % (95 % CI 99.1 %–100 %). The prevalence of the most abundant tick species per sanctuary was highly variable, ranging from 100 (80.5 − 100) for *A*. *gemma* in NRS to 26.3 (13.4 − 43.1) for *H*. *truncatum* in OLJ (Table 7). For Sanctuaries with white and black rhinoceros in sympatry, chi-square test revealed a lack of association between prevalence of the different tick species and the host rhinoceros species (***χ*^*2*^**_*1*_ = 0.087, p = 0.768, Fig. 6). Given the low number of white rhinoceros sampled in several sanctuaries, only the combined prevalence is therefore presented (Table 8). The mean number of ticks parasitizing a rhinoceros was on average (mean + SD) of 38.53 + 40.59 ticks per host with notable variation across rhinoceros sanctuaries. Hosts were infested with up to eight species of ticks simultaneously with an average (mean ± sd) of 3.45 ± 1.49 species infesting a single rhinoceros. Tick infestation patterns examined for the 3 most abundant tick species in each rhinoceros population or for all tick species combined displayed a wide range of aggregation patterns as indicated by two thirds of the tick species across rhinoceros sanctuaries having K less than 1 and Poulin's D ranging from 0.203 to 0.844 (Table 8).

**Table 7:** Tick prevalence and abundance for 2 to 4 dominant ticks including all ticks combined in each rhinoceros Sanctuary.

Sanctuary	Tick species	Infested rhinoceros	Rhinoceros examined NSame as above	Prevalence (95 % LCL-UCL)	Mean abundance (95 % LCL-UCL)
IPZ	*R. humeralis*	13	25	52.0 (31.3 − 72.2)	30.65 (17.6 − 46.8)
	*A. gemma*	24	25	96.0 (79.6 − 99.9)	20.4 (15.0 − 26.9)
	*A. sparsum*	19	25	76.0 (54.9 − 90.6)	9.60 (6.16 − 13.0)
	All species	25	25	100.0 (86.3 − 100)	78.10 (52.3 − 105)
LBL	*A. gemma*	105	112	93.8 (87.5 − 97.5)	12.6 (10.2 − 15.5)
	*R. pulchellus*	98	112	87.5 (79.9–93.0)	11.8 (9.38 − 14.7)
	*A. sparsum*	81	112	72.3 (63.1 − 80.4)	14.9 (11.6 − 19.4)
	All species	112	112	100.0 (96.8 − 100)	48.1 (40.9 − 56.8)
LNP	*A. rhinocerotis*	19	23	82.6 (61.2 − 95)	6.39 (3.52 − 16.4)
	*A. variegatum*	18	23	78.3 (56.3 − 92.5)	8.3 (4.43 − 17.6)
	*A. sparsum*	7	23	30.4 (13.2–52.9)	1.39 (0.48 − 3.00)
	All species	23	23	100 (85.2 − 100)	20 (9.74 − 41.2)
MNP	*A. gemma*	21	23	91.3 (72 − 98.9)	18.3 (13.4 − 24.7)
	*R. pulchellus*	17	23	73.9 (51.6 − 89.8)	9.48 (2.91 − 24.5)
	*A. tholloni*	11	23	47.8 (26.8 − 69.4)	1.39 (0.696 − 2.39)
	All species	23	23	100 (85.2 − 100)	35.9 (21.5 − 59)
MNR	*A. cohaerens*	25	26	96.2 (80.4 − 99.9)	28.40 (20.9 − 36.2)
	*R. praetextatus*	14	26	53.8 (33.4 − 73.4)	3.81 (2.14 − 5.81)
	*A. sparsum*	14	26	53.8 (33.4 − 73.4)	1.65 (0.92 − 2.73)
	*A. tholloni*	11	26	42.3 (23.4 − 63.1)	1.62 (0.65 − 4.22)
	All species	26	26	100 (86.8 − 100)	36.70 (27.0 − 46.8)
NNP	*A. gemma*	23	30	76.7 (57.7 − 90.1)	5.83 (3.53 − 10.1)
	*A. rhinocerotis*	11	30	36.7 (19.9 − 56.1)	2.87 (1.00 − 9.73)
	*R. pulchellus*	26	30	86.7 (69.3 − 96.2)	5.73 (3.80 − 8.27)
	*A. variegatum*	18	30	60 (40.6 − 77.3)	2.33 (1.27 − 3.77)
	*A. sparsum*	24	30	80 (61.4 − 92.3)	4.90 (3.20 − 7.96)
	All species	30	30	100 (88.4 − 100)	25.50 (19.9 − 40.7)
NRS	*A. gemma*	17	17	100 (80.5 − 100)	28.60 (21.8 − 35.8)
	*A. sparsum*	14	17	82.4 (58.3 − 95)	19.01 (13.2 − 23.6)
	*R. humeralis*	13	17	76.5 (50.1 − 93.2)	11.50 (7.40 − 17.3)
	All species	17	17	100 (80.5 − 100)	77.4 (56.8 − 96.7)
OLJ	*A. gemma*	32	38	84.2 (68.7 − 94)	7.79 (5.54 − 10.7)
	*A. sparsum*	25	38	65.8 (48.6 − 80.4)	2.95 (2.11 − 4.13)
	*R. pulchellus*	30	38	78.9 (62.7 − 90.4)	13.7 (9.55 − 19.5)
	*H. truncatum*	10	38	26.3 (13.4 − 43.1)	2.74 (1.08 − 6.50)
	All species	38	38	100 (90.7 − 100)	27.3 (21.9 − 35.1)
OPC	*A. sparsum*	49	55	89.1 (77.8 − 95.9)	8.96 (6.4 − 13.4)
	*A. tholloni*	45	55	81.8 (69.1 − 90.9)	3.87 (2.91 − 5.07)
	*R. pulchellus*	37	55	67.3 (53.3 − 79.3)	4.78 (3.24 − 7.33)
	All species	55	55	100 (93.5 − 100)	19.3 (14.9–26.0)

Lower Confidence Level (LCL) ─ Upper Confidence Level (UCL).

**Table 8:** Tick aggregation patterns expressed using Variance to mean ratio, Poulin's Discrepancy Index and dispersion parameter K for 2 to 4 dominant ticks including all tick species combined in each rhinoceros sanctuary.

Sanctuary	Tick species	Variance/mean ratio	Poulin's Discrepancy Index (LCL -UCL)	Neg- binomial exponent K	p-value
IPZ	*R. humeralis*	43.61	0.60 (0.47 − 0.75)	0.176	NA
	*A. gemma*	11.04	0.39 (0.32 − 0.49)	1.567	0.162
	*A. sparsum*	8.1	0.49 (0.35 − 0.63)	0.614	0.128
	All species	61.73	0.46 (0.35 − 0.57)	0.977	0.064
LBL	*A. gemma*	16.88	0.575 (0.533 − 0.621)	0.7925	0.2435
	*R. pulchellus*	17.13	0.613 (0.569 − 0.661)	0.6572	0.1068
	*A. sparsum*	29.52	0.663 (0.609 − 0.726)	0.3541	0.0311
	All species	37.88	0.477 (0.43 − 0.526)	1.1293	0.1579
LNP	*A. rhinocerotis*	21.96	0.616 (0.437 − 0.77)	0.6363	0.6365
	*A. variegatum*	22.92	0.637 (0.528 − 0.767)	0.5282	0.7595
	All species	57.04	0.62 (0.531 − 0.699)	0.7557	0.038
MNP	*A. gemma*	10.97	0.409 (0.323 − 0.538)	1.2451	0.9648
	*R. pulchellus*	51.84	0.779 (0.72 − 0.86)	0.2899	0.040
	*A. tholloni*	2.99	0.667 (0.542 − 0.803)	0.5209	NA
	All species	54.06	0.534 (0.464 − 0.655)	0.9486	0.600
MNR	*A. cohaerens*	15.02	0.39 (0.29 − 0.51)	1.260	0.099
	*R. praetextatus*	5.82	0.61 (0.48 − 0.75)	0.366	0.422
	*A. sparsum*	3.24	0.65 (0.54 − 0.79)	0.598	0.995
	*A. tholloni*	9.56	0.78 (0.68 − 0.89)	0.270	0.362
	All species	9.56	0.78 (0.68 − 0.89)	0.270	0.362
NNP	*A. gemma*	12.57	0.635 (0.549 − 0.752)	0.552	0.7301
	*A. rhinocerotis*	24.77	0.843 (0.773 − 0.92)	0.145	0.8966
	*R. pulchellus*	6.9	0.534 (0.46 − 0.64)	0.9683	0.9341
	*A. variegatum*	4.98	0.662 (0.575 − 0.769)	0.5185	0.864
	*A. sparsum*	7.53	0.56 (0.46 − 0.688)	0.822	0.99
	All species	22.49	0.382 (0.279 − 0.499)	2.1613	0.1707
NRS	*A. gemma*	8.53	0.28 (0.20 − 0.38)	3.372	0.212
	*A. sparsum*	6.59	0.29 (0.16 − 0.50)	1.120	NA
	*R. humeralis*	10.27	0.46 (0.34 − 0.66)	0.746	0.558
	All species	23.06	0.28 (0.19 − 0.42)	2.360	0.291
OLJ	*A. gemma*	3.63	0.56 (0.46 − 0.68)	0.797	0.544
	*A. sparsum*	7.77	0.50 (0.41 − 0.60)	0.964	0.250
	*R. pulchellus*	17.63	0.56 (0.48 − 0.66)	0.591	0.425
	*H. truncatum*	20.36	0.85 (0.78 − 0.92)	0.092	0.149
	All species	16.21	0.40 (0.33 − 0.48)	1.604	0.282
OPC	*A. sparsum*	18.43	0.596 (0.524 − 0.682)	0.7796	0.1406
	*A. tholloni*	4.5	0.538 (0.475 − 0.621)	1.0878	0.636
	*R. pulchellus*	11.25	0.666 (0.582 − 0.741)	0.4503	0.7149
	All species	20.04	0.484 (0.436 − 0.536)	1.393	0.0002

95 % Lower Confidence Level (LCL)─Upper Confidence Level (UCL).

**Fig. 7:**
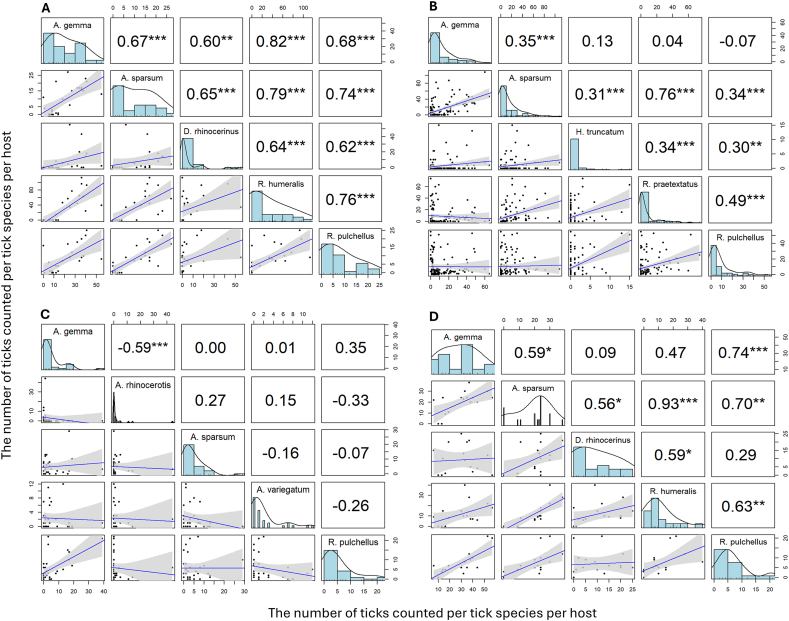
Correlations between tick species across individual hosts at: (A). IPZ, (B). LBL, (C). NNP and (D). NRS indicating positive pairwise correlations in tick species abundance among individual hosts.

There were moderate to high positive and statistically significant correlations among abundant ticks across individual hosts at IPZ, and NRS and low positive and statistically significant correlations at LBL (Fig. 7). There were six out of ten low and negative correlations among ticks in NNP but only one was statistically significant.

Generalized Linear Mixed Models revealed that among the environmental factors influencing tick abundance, mean daily temperature metrics was the most important variable, and was a predictive variable in all species examined. Generally, the abundance of most ticks was positively influenced by average daily minimum temperatures (*H. truncatum, A. gemma*, *A. tholloni, A. cohaerens,* and *A. sparsum*) and mean daily average temperatures (*H. rufipes)* respectively (Table 9). On the other hand, the abundance of *R. pulchellus,* and *R. praetextatus* were negatively influenced by mean daily maximum temperature whereas *A. variegatum* and *A. rhinocerotis* were negatively influenced by mean daily average and mean daily minimum temperatures respectively. NDVI (Normalized Difference Vegetation Index) was a predictor of tick abundance in 8 of 10 tick species evaluated. Specifically, monthly NDVI positively influenced the abundance of *A. gemma*, *A. sparsum*, *A. variegatum*, *R. pulchellus A. rhinocerotis,* and *A. tholloni* and negatively influenced the abundance of *R. praetextatus*, and *H. truncatum*. Spatial heterogeneity in NDVI positively influenced the abundance of *A. cohaerens* and *A. rhinocerotis* but negatively influenced the abundance of *A. gemma*, *R. praetextatus,* and *A. sparsum* (Table 9).

**Table 9:** Best models showing the influence of Rainfall, Temperature, Normalized Difference Vegetation Index (monthly values), infestation by other tick species and host factors (Sex, Age and rhinoceros species) on the abundance of different tick species infesting rhinoceros across their range in Kenya.

Tick Species	Variables	Estimate	Std. error	z value	P(>|z|)
*A. cohaerens*	Intercept	−15.517	5.475	−2.83	0.0046
	Sex (male cf. female)	0.195	0.078	2.50	0.0125
	Age in years	−0.196	0.041	−4.75	<0.0001
	Mean spatial NDVI	1.183	0.145	8.18	<0.0001
	Mean daily minimum temperature	0.285	0.037	7.75	<0.0001
	Abundance of other tick species	0.531	0.195	2.73	0.0063
*A. gemma*	Intercept	−0.287	0.878	−0.33	0.7437
	Age in years	0.799	0.050	15.93	<0.0001
	Mean NDVI	0.416	0.030	13.76	<0.0001
	Mean spatial NDVI	−0.132	0.042	−3.11	0.0019
	Total monthly rainfall	0.188	0.049	3.80	0.0001
	Mean daily minimum temperature	0.415	0.074	5.62	0.0000
	Abundance of other tick species	0.213	0.018	12.19	<0.0001
*A. sparsum*	Intercept	0.007	0.792	0.01	0.9931
	Sex (male cf. female)	0.215	0.038	5.73	<0.0001
	Rhinoceros species (white cf. black)	0.195	0.046	4.21	<0.0001
	Age in years	−0.132	0.020	−6.64	<0.0001
	Mean NDVI	0.428	0.028	15.16	<0.0001
	Mean spatial NDVI	−0.058	0.037	−1.56	0.1199
	Total monthly rainfall	−0.146	0.045	−3.27	0.0011
	Mean daily minimum temperature	0.945	0.127	7.42	<0.0001
	Abundance of other tick species	0.481	0.020	24.27	<0.0001
*A. tholloni*	Intercept	−2.599	1.010	−2.57	0.0101
	Sex (male cf. female)	−0.215	0.117	−1.84	0.0661
	Rhinoceros species (white cf. black)	−0.478	0.165	−2.90	0.0038
	Age in years	0.253	0.056	4.52	<0.0001
	Total monthly rainfall	0.662	0.179	3.70	0.0002
	Mean daily minimum temperature	1.004	0.196	5.12	<0.0001
	Abundance of other tick species	−0.235	0.103	−2.29	0.0219
*A. rhinocerotis*	Intercept	−3.950	1.295	−3.05	0.0023
	Sex (male cf. female)	0.270	0.114	2.36	0.0181
	Rhinoceros species (white cf. black)	−1.295	0.216	−5.99	<0.0001
	Age in years	0.520	0.075	6.89	<0.0001
	Mean NDVI	1.054	0.102	10.29	<0.0001
	Standard Deviation of spatial NDVI	0.508	0.125	4.05	0.0001
	Total monthly rainfall	−0.384	0.109	−3.54	0.0004
	Mean daily minimum temperature	−2.932	0.349	−8.41	<0.0001
*A. variegatum*	Intercept	−4.812	1.196	−4.03	0.0001
	Sex (male cf. female)	0.276	0.141	1.95	0.0511
	Rhinoceros species (white cf. black)	1.182	0.177	6.68	<0.0001
	Age in years	−0.540	0.140	−3.86	0.0001
	Mean NDVI	1.346	0.102	13.23	<0.0001
	Total monthly rainfall	−1.267	0.125	−10.10	<0.0001
	Mean daily average temperature	−0.578	0.138	−4.20	<0.0001
*R. pulchellus*	Intercept	0.587	0.997	0.59	0.5560
	Sex (male cf. female)	−0.288	0.040	−7.25	<0.0001
	Rhinoceros species (white cf. black)	−0.219	0.048	−4.55	<0.0001
	Age in years	0.116	0.018	6.63	<0.0001
	Mean NDVI	0.239	0.027	8.80	<0.0001
	Mean daily maximum temperature	−0.804	0.068	−11.73	<0.0001
	Abundance of other tick species	0.337	0.020	16.71	<0.0001
*R. praetextatus*	Intercept	−0.694	0.790	−0.88	0.3800
	Sex (male cf. female)	0.465	0.057	8.19	<0.0001
	Rhinoceros species (white cf. black)	−0.577	0.065	−8.88	<0.0001
	Age in years	0.276	0.029	9.44	<0.0001
	Mean NDVI	−0.260	0.036	−7.29	<0.0001
	Standard Deviation of spatial NDVI	−0.879	0.068	−12.91	<0.0001
	Mean daily maximum temperature	−0.510	0.088	−5.80	<0.0001
	Abundance of other tick species	0.589	0.027	21.80	<0.0001
*H. truncatum*	Intercept	−3.722	1.179	−3.16	0.0016
	Rhinoceros species (white cf. black)	1.356	0.198	6.84	<0.0001
	Age in years	0.440	0.087	5.03	<0.0001
	Mean NDVI	−0.451	0.112	−4.05	0.0001
	Mean daily minimum temperature	1.124	0.365	3.08	0.0021
	Abundance of other tick species	0.809	0.072	11.20	<0.0001
*H. rufipes*	Intercept	−3.025	0.581	−5.21	<0.0001
	Sex (male cf. female)	0.574	0.190	3.02	0.0025
	Mean daily average temperature	1.232	0.319	3.86	0.0001
	Abundance of other tick species	0.490	0.101	4.84	<0.0001

The total monthly rainfall served as a significant predictor of abundance for half of the species examined, although its impact differed among them. Rainfall negatively influenced the abundance of most adult ticks of *A*. *sparsum*, *A. rhinocerotis, A. variegatum,* and *R. praetextatus* but had a positive effect on the abundance of *A. gemma,* and *A. tholloni*.

Age of the rhinoceros was the most important host variable influencing tick abundance (9 in 10 tick species). Tick abundance was positively related to age in *A. gemma, A. tholloni*, *A. rhinocerotis, R. pulchellus*, *R. praetextatus* and *H. trancutum*, but negatively related to age in *A. cohaerens*, *A. sparsum,* and *A. variegatum*.

Among the host factors driving abundance of specific tick species, the abundance of other tick species was important in 8 of 10 tick species evaluated. The abundance of *A. cohaerens*, *A. gemma*, *A. sparsum. R. pulchellus, R. praetextatus*, *H. truncatum* and *H. rufipes* were positively influenced by the abundance of other tick species, while *A. tholloni* was negatively influenced by the abundance of other tick species considered.

Sex of the host rhinoceros was an important predictor of tick abundance in 8 of ten tick species*. The abundance of A. cohaerens, A. sparsum, A. rhinocerotis, A. variegatum, R. praetextatus*, and *H. rufipes* higher in males compared to females but abundance of *A. tholloni* and *R. pulchellus* were higher in females compared to males (Table 9).

Rhinoceros species was also an important predictor in seven of ten species with abundance of *A. sparsum*, *A. variegatum*, and *H. truncatum* higher in white rhinoceros, compared to black rhinoceros. The abundance of *A. tholloni*, *A. rhinocerotis*, *R. pulchellus*, and *R. praetextatus* were significantly lower in white rhinoceros compared to black rhinoceros.

## 4 Discussion

### 4.1 Tick communities and the influence of NDVI, rainfall and temperature

Ticks within the Kenyan rhinoceros meta-population had pronounced heterogeneity in tick species composition, with the twelve sanctuaries examined forming six communities. There was substantial similarity in tick composition within communities. Tick species composition was unique for rhinoceros from MNR and SER, with tick infestations dominated by *A*. *cohaerens*, and *H*. *rufipes*, respectively. In support, Walker (1974) reported that populations of *A. cohaerens* in Kenya prefer sub-humid and semi-arid ecological zones but their distribution is limited to Narok (where MNR is located) and Nyanza regions, while *H. rufipes* was reported to prefer arid and semi-arid ecological zones with a much wider distribution. IPZ and NRS formed a single community of tick species comprising some ticks with limited distribution like *D. rhinocerinus* and *R. humeralis* both restricted to the sanctuaries in the Tsavo Ecosystem. Historical records reveal that *D. rhinocerinus* and *R. humeralis* ticks prefer the arid ecological zone and geographical distribution restricted to the southern districts of the eastern and coastal province of Kenya where the Tsavo ecosystem lies (Walker, 1974). IPZ and NRS also have some widespread tick species including *A. gemma* and *A. sparsum*. Sanctuaries like OPC, LBL and NNP had a similar composition of tick species, including *A*. *sparsum* and *R*. *pulchellus* among the dominant ticks. Walker (1974) reports that *A. sparsum* is found in a wide range of ecological zones in Kenya from dry sub-humid to semi-arid ecological zones while *R. pulchellus* inhabits the semi-arid to arid ecological zones located in the dry eastern parts of the rift valley. LNP and SRS had similar tick species composition largely characterized by *A. rhinocerotis* and *R. praetextatus* among some of the dominant tick species. Historical distribution of *A. rhinocerotis* shows it is common in the southern half of the eastern province, including Meru, Embu and the Tsavo ecosystem and its most common in the arid ecological zone but also survives at the fringes humid to semi-arid ecological zones (Walker, 1974) while *R*. *praetextatus* occurs in a wide range of ecological conditions, from semi-arid habitats through tropical and subtropical savanna to wet wooded highland areas (Walker et al., 2000). The tick species composition in TEN, OLJ, and MNP were similar forming a single tick community dominated by species widely distributed in arid and semi-arid regions, especially *A. gemma* and *R. pulchellus*.

The distinct tick communities appeared to be shaped by mean NDVI, and temperature metrics. Mean NDVI was explained 14.87 % of variation in tick species composition across sanctuaries. After addressing collinearity among mean temperature, minimum temperature and maximum temperature into a model containing mean NDVI, 60.48 % of the total variance in the relative composition of tick species was explained. Mean NDVI is a proxy for vegetation health and density, which in turn relates to primary productivity, moisture, and microclimatic conditions that influence tick survival (Cihlar et al., 1991; De et al., 2024). In addition, mean NDVI influences the distribution and abundance of large herbivore species (hosts) in Kenya, as regions with high interannual average NDVI tend to have higher host species richness and ecoclimatic stability (Oindo and Skidmore, 2002). The presence and movement of these hosts directly impact the abundance and distribution of different tick species. Minimum temperature, mean temperature and maximum temperature are often better predictors of tick species distribution because ticks are highly sensitive to desiccation, and moisture availability (linked to rainfall and vegetation cover) is a critical limiting factor for their survival (Fieler et al., 2021).

The observed heterogeneity in species composition across sanctuaries reveals the role of historical distributions and contemporary ecological conditions (mean NDVI, and temperature), explains a substantial portion of the variance in tick species composition, highlighting the importance of vegetation health and climate as key determinants of tick survival and distribution. The primary limitation of this study is its inability to distinguish between the influence of similar environments and geographic proximity (distance decay) in nearby tick communities (Warburton et al., 2016).

### 4.2 A comparison of tick diversity with other populations and animal hosts

Kenyan rhinoceros exhibit exceptional tick species richness — 20 species across four genera—far surpassing the diversity observed in South African rhinoceros (7 species, 4247 ticks, from 381 rhinoceros), Kenya's elephants (8 species, 1964 ticks from 128 elephants), and even a heavily infested cattle population in Karamoja Uganda (15 species, 17,562 ticks collected from 1531 cattle) (Horak et al., 2017; Kariuki et al., 2019; Etiang et al., 2024). This richness is comparable to that of the common eland which harbored 21 tick species identified from 36,693 adult ticks collected from 36 hosts from various locations in South Africa (Horak et al., 2007).

Diversity indices such as Hill-Shannon and Hill-Simpson further mirror differences observed for tick species richness. Compared to other large mammals Kenya's rhinoceros had a high tick species diversity. Data presented by Kariuki et al. (2019), on ticks parasitizing elephants from 60 locations in Kenya had an asymptotic Hill-Shannon and Hill-Simpson estimates of 2.70 and 2.06 respectively. Moreover, a study on cattle in the Karamoja region of Uganda by Etiang et al. (2024) revealed asymptotic Hill-Shannon and Hill-Simpson estimates at 4.31 and 3.50 respectively. The diversity of ticks infesting the rhinoceros in Kenya was however similar to that of eland in South Africa, which had an estimated Hill-Shannon and Hill-Simpson diversity of 8.03 (5 % CI: 7.99–8.06) and 6.27 (95 % CI:6.24–6.32).

The variation between tick species richness and diversity between Kenya and South Africa rhinoceros could be related to the regional variation in tick species diversity which is known to follow a latitudinal gradient with the highest species richness in east equatorial Africa and lowest in higher latitudes like South Africa (Cumming, 2000). The relative variation in tick species diversity between different host species although known to be strongly influenced by body size does not apply in this case as the African elephant with large body size and occupying the same habitats as the rhinoceros had lower tick species diversity. This may be related to host variation in chemical signatures of odorants that attract ticks to host (Donzé et al., 2004; Long et al., 2023; Bezerra-Santos et al., 2024). This result highlights the value of rhinoceros as sentinels for studies on ixodid tick diversity.

### 4.3 Intrapopulation variation in tick species richness and diversity

There was significant variation in empirical species richness and diversity metrics among the twelve rhinoceros sanctuaries examined. Species richness was notably high for IPZ (13), followed closely by NNP (10), and OPC (10) and significantly lower in SER, which had only 2 species. The Hill-Shannon and Hill-Simpson diversity metrics followed similar patterns with NNP (6.59 and 5.80 respectively), IPZ (4.91 and 3.91) and NRS (4.88 and 4.14) having higher values. On the lower end of the spectrum, SER (1.83, 1.69), MNR (2.40, 1.63) and MNP (3.58, 2.83), had the lowest Hill-Shannon and Hill-Simpson diversity metrics.

In this study, tick species richness and diversity were significantly influenced by spatial heterogeneity in NDVI and to a lesser degree maximum temperature and mean NDVI. A positive relationship between the log of species richness and spatial heterogeneity in NDVI and tick burden was observed at the level of the sanctuary. At the host level, however, monthly mean NDVI and monthly spatial heterogeneity in NDVI were positively correlated with tick species richness while monthly maximum temperature negatively influenced tick species' richness. However, when the number of ticks infesting an individual animal was included in the model along with other covariates, tick species richness was driven only by spatial heterogeneity in NDVI alone. NDVI, both in terms of mean monthly values and spatial heterogeneity, correlate positively with tick species richness, perhaps because denser and more varied vegetation supports a greater diversity of microhabitats and potential hosts (Oindo and Skidmore, 2002; Estrada-Peña et al., 2004; Chidodo et al., 2020; Mutizhe et al., 2021). Studies elsewhere have revealed a pivotal role of environmental drivers (temperature, rainfall, humidity) in shaping tick species richness and diversity (Cumming, 2002; Dantas-Torres and Otranto, 2013; Okabe et al., 2022). Studies on thermal tolerance of ixodid tick larvae, (Leal et al., 2020; Fieler et al., 2021), have demonstrated that the larvae of the majority of ixodid tick species have preferred or optimal temperatures between 17 °C and 22 °C (Fieler et al., 2021) above or below which their survival growth and development can be compromised. For tropical ticks, the lower and upper temperature tolerance limits for tick development range between 15 °C and 37 °C (Punyua, 1992). The mean monthly temperatures for most rhinoceroses’ sanctuaries appear to meet the global larval optimum temperature suitable for tick development and survival.

These findings highlight the importance of environmental factors in shaping tick communities and underscore the value of targeted studies for identifying tick diversity hotspots and the evolution of vector-pathogen dynamics.

### 4.4 Host infestation patterns: tick aggregation and abundance

In this study, aggregation indices (K < 1 and Poulin's D ranging from 0.203 to 0.844) mirror patterns observed in other large mammals, as well as in birds and fish. Two-thirds of tick species exhibited strong aggregation (K < 1), indicating that a small subset of hosts harbored disproportionately large tick burdens. The degree of tick aggregation on hosts has important implications for tick borne diseases (TBD) transmission, persistence and establishment in any locality and is vital for understanding TBD epidemiology (Randolph et al., 1999; Harrison and Bennett, 2012) through co-feeding. The aggregation parameter closely mirrors observations on parasites in other mammals, fish and birds. The aggregation parameter (K) for *Dermacentor reticulatus* on two rodents, field mouse, *Apodemus falvicollis* and the bank vole, *Cleithrionomys glareolus* was found to range from 0.13 to 0.284 in two localities in Slovakia (Randolph et al., 1999). K for the tick, *Ixodes ricinus* parasitizing the great tit (*Parus major*) was 0.26 (Heylen et al., 2013). In a study of helminth parasite aggregation, the aggregation parameter (K) for the helminth *Discocotyle sagittata* infecting rainbow trout, *Oncorhynchus mykiss,* was estimated to be 0.64 indicating strong parasite aggregation (Tinsley et al., 2020).

Multi-species infestations were common, with rhinoceros typically hosting on average three tick species and up to eight tick species simultaneously. Positive correlations in tick coinfection patterns suggest that individual host-specific factors are a primary driver in determining an individual's overall exposure and susceptibility to infection. Increased susceptibility of some individuals can potentially arise due to genetic, behavioral, or physiological factors (Owen et al., 2025). Correlation analyses among abundant tick species highlighted strong positive relationships in some sanctuaries (IPZ, NRS, LBL), suggesting shared environmental or host-related drivers of co-infestation, while other locations exhibited weaker or non-significant correlations, possibly reflecting competitive exclusion or habitat differentiation among tick species. However, a single pair of tick species in NNP showed a moderate and statistically significant negative correlation. These observations suggest that factors influencing tick aggregation vary by location with individual host factors (grooming and wallowing behaviors, immune susceptibility, sex, age and species) being dominant and tick competition being important in a few rare cases (Brehm et al., 2025).

### 4.5 Factors influencing variation in tick abundance between hosts

Environmental variables such as temperature, NDVI, and rainfall, along with host characteristics like age, sex, and species, were significant predictors of tick abundance. Monthly temperature, NDVI, and its’ spatial heterogeneity were significant environmental predictors for the abundance of various tick species. The abundance of *H. trancutum*, *H. rufipes*, *A. sparsum*, *R. pulchellus*, *A. gemma*, *A. variegatum*, *A. cohaerens*, and *R. praetextatus* were positively associated with mean monthly temperature but negatively associated with temperature for *A. rhinocerotis* and *A. tholloni*.

Temperature significantly impacts the abundance of engorged ticks in all tick species examined. Average daily minimum temperatures positively influence the abundance of *H. truncatum, A. gemma*, *A. tholloni, A. cohaerens,* and *A. sparsum* while mean daily average temperatures positively influenced the abundance of *H. rufipes)*. The abundance of *R. pulchellus,* and *R. praetextatus* were negatively influenced by mean daily maximum temperature whereas *A. variegatum* and *A. rhinocerotis* were negatively influenced by mean daily average and mean daily minimum temperatures respectively. Warm temperatures generally accelerate tick development, leading to a faster life cycle and potentially higher reproductive rates (Nuttall, 2022). Conversely, colder temperatures can prolong the life cycle, reducing the overall tick density (Eisen et al., 2016). While moderate warmer temperatures generally increase tick survival and activity while extremely high temperatures can lead to dehydration and mortality. Furthermore, tick populations can be influenced by density-dependent factors, meaning that high tick populations may lead to increased mortality rates due to competition and resource scarcity (Ogden and Lindsay, 2016).

NDVI is a proxy for vegetation cover, and a higher NDVI generally indicates more suitable habitats for ticks. Ticks, particularly nymphs and adults, often prefer areas with dense vegetation, which provides them with shelter from extreme temperatures and precipitation, as well as potential hosts. A higher NDVI can mean that there is more shade and moisture retention, which can positively influence the number of ticks questing. Furthermore, the presence of vegetation can also influence the types of host animals available, potentially increasing the availability of potential blood-meals for ticks. For example, in arid areas in Tanzania NDVI was strongly and positively correlated with rodent abundance (Chidodo et al., 2020) the major hosts of the larvae and nymphs of most of the three host ticks infesting rhinoceros. NDVI was a predictor of tick abundance in 7 of 10 tick species evaluated. Specifically, monthly NDVI positively influenced the abundance of *A. gemma*, *A. sparsum*, *A. variegatum*, *R. pulchellus* and negatively influenced the abundance of *A. rhinocerotis*, *R. praetextatus*, *H. truncatum*.

Spatial heterogeneity in NDVI, a measure of vegetation spatial heterogeneity, positively influenced the abundance of *A. sparsum* and *R. pulchellus* but was negatively correlated with the abundance of *A. gemma*, *A. rhinocerotis*, *A. variegatum*, and *R. praetextatus*.

Monthly rainfall totals negatively influenced the abundance of most adult ticks in rhinoceros (*A. cohaerens*, *A. gemma*, *A. sparsum*, *A. variegatum*, *R. pulchellus*) but had a positive effect on the abundance of *A. tholloni*. While rainfall can have a positive impact on tick populations in some areas (Mooring et al., 1994; Keesing et al., 2018), especially those with lower humidity, it can also have negative effects in arid areas. Rainfall can increase humidity, providing a more favorable environment for ticks, especially in areas where humidity is a limiting factor. This can lead to increased tick activity, including questing behavior, and potentially higher abundance of engorged adults. In arid or semi-arid areas, excessive rainfall can be detrimental to tick populations. Prolonged periods of wetness can lead to drowning or other negative impacts on tick development and survival (Weiler et al., 2017), potentially reducing the number of engorged adults.

Age can influence the abundance of engorged ticks, but the specific relationships vary between tick species and host animals. In some cases, adult animals may have higher tick burdens due to longer exposure and changes in immunity and variation in body size with age. Adults have lived for a longer period and have been exposed to ticks more frequently. In some cases, older animals may have weakened their immune systems, making them more vulnerable to tick infestations. In 7 out of 9 tick species showing age effects (*H. trancutum, R. pulchellus*, *R. praetextatus*, *A. gemma*, *A. sparsum, A. tholloni*, *A. rhinocerotis*), tick abundance increased with age and only two (*A. cohaerens* and *A. variegatum*) did tick abundance decrease with age.

Host sex can influence tick abundance and infestation patterns. Males have been found to have higher tick burdens than females in several studies perhaps resulting from sex variation in activity pattern, with males in territorial or polygynous species like rhino being more active and explore larger areas of their territories, increasing their risk of encountering ticks. In this study, 5 of 10 tick species examined, sex was an important predictor of abundance, with males being more parasitized by *A. cohaerens*, *A. variegatum*, *R. praetextatus*, *H. truncatum* and *H. rufipes* as compared to females. This pattern of male biased tick parasitism has been observed in red deer (Ruiz-Fons et al., 2013), roe deer (Kiffner et al., 2011), and spur-thighed tortoise (Segura et al., 2023). The contrast has been observed in cattle from different locations, where females had heavy tick infestation than males (Jemal et al., 2021; Khan et al., 2022). There were no females preferentially parasitized by any tick species in this study. Mating system (Miller et al., 2007), sexual dimorphism (Moore and Wilson, 2002) intense intraspecific competition among males relative to females (Bacelar et al., 2011), testosterone levels (Hughes and Randolph, 2001) have been proposed as relevant factors driving sex-biased parasitism in mammals.

### 4.6 Pathogen transmission potential: conservation implications

This research contributes to understanding on the ecology of rhinoceros ticks, their prevalence, distribution and abundance across sub populations in Kenya. The competent tick vectors of *Theileria bicornis* and *Babesia bicornis*, two pathogens that have known to threaten the African Rhinoceros conservation are not known. However, a recent study in Kenya, detected *Theileria bicornis* in adult *Amblyomma tholloni* ticks infesting African elephants suggesting they are potential vectors for this rhinoceros pathogen (King'ori et al., 2019). In a study on the epidemiology *T. bicornis*, in six Kenyan rhinoceros sub populations, revealed the widespread distribution of *Theileria bicornis* in the Kenyan rhinoceros metapopulations with 49.12 % prevalence and the presence of infections in all the sub populations examined (Otiende et al., 2015). In two of the four locations LNP, MNP, NNP, NRS, which overlapped with this study, there was no *Amblyomma tholloni* recorded in LNP and NRS perhaps because the tick populations were too low to be detected or the tick species was simply absent in these locations.

In the Ngorongoro Crater, Tanzania, *Anaplasma marginale* was detected in 6 tick species including some of the most abundant ticks infesting rhinoceros in this study such as *Amblyomma gemma*, *R. praetextatus* and *R. pulchellus* (Penzhorn et al., 2008). *Anaplasma* sp has also been detected to infect rhinoceros (Makgabo et al., 2023). *Amblyomma gemma, Amblyomma sparsum* and *Amblyomma variegatum*, are known vectors for several pathogens, most notably *Ehrlichia ruminantium*, the agent of heartwater disease in ruminants, and the zoonotic *Rickettsia africae*, the agent of African tick-bite fever (Walker and Olwage, 1987; Wesonga et al., 2001). Serological evidence suggest Rhinoceros are susceptible to infection by *Ehrlichia ruminatum* in south Africa (Kock et al., 1992). Other vectors also include *A. cohaerens*, and *A. tholloni* (Peter et al., 2000).

The detection of multiple tick-borne pathogens, including *Anaplasma marginale*, *Ehrlichia ruminantium*, and *Rickettsia africae*, in tick species that commonly infest Kenyan rhinoceroses has significant implications for the conservation and health management of rhinoceros populations. The presence of these pathogens in both ticks and rhinoceros hosts highlights the ongoing risk of disease transmission, which can adversely affect rhinoceros health and potentially lead to morbidity or mortality events. The fact that competent vectors such as *Amblyomma gemma*, *Amblyomma sparsum*, and *Amblyomma variegatum* are widespread and abundant increases the likelihood of pathogen circulation within and between populations. These observations highlight the need for surveillance of these pathogens in Kenyan ticks and rhinoceros under a One Health approach—integrating wildlife, livestock, and human health perspectives—to safeguard both endangered rhinoceros and public health. Prioritizing research on tick-pathogen-host dynamics in Kenyan rhinoceros habitats will be crucial for developing effective, evidence-based conservation and disease management policies.

The findings of this study advance our understanding of tick ecology in Kenyan rhinoceroses’ populations and offer essential baseline data for future conservation and health management initiatives. The high species richness and diversity, coupled with strong aggregation and sanctuary-specific assemblages, highlight the need for site-specific tick control measures and disease surveillance programs. Environmental factors, particularly vegetation heterogeneity and temperature, are central to shaping tick communities and should inform habitat management and restoration efforts. Host-related factors, while secondary, also warrant consideration in population management and translocation planning. Importantly, the identification of vector species with potential to transmit TBPs underlines the necessity of integrating tick and pathogen monitoring into broader rhinoceros conservation strategies. Targeted interventions, informed by ecological and epidemiological data, will be critical in mitigating tick-borne disease risks and promoting the long-term survival of rhinoceros populations in Kenya.

## CRediT authorship contribution statement

**Edward M. King'ori:** Writing – review & editing, Writing – original draft, Project administration, Methodology, Investigation, Formal analysis, Data curation, Conceptualization. **Patrick I. Chiyo:** Writing – review & editing, Writing – original draft, Visualization, Validation, Investigation, Formal analysis, Data curation, Conceptualization. **Olgabeth N. Gitau:** Writing – review & editing, Writing – original draft, Validation, Methodology, Investigation, Conceptualization. **Fredrick Lala:** Writing – review & editing, Writing – original draft, Visualization, Validation, Supervision, Methodology, Data curation, Conceptualization. **Olivia Wesula Lwande:** Writing – review & editing, Writing – original draft, Visualization, Validation, Supervision, Resources, Investigation, Funding acquisition, Formal analysis, Conceptualization.

## Conflict of interest statement

The authors declare that they have no known competing financial interests or personal relationships that could have appeared to influence the work reported in this paper.
