# Supplementary material for: Drivers of tick community structure in a rhinoceros meta-population in Kenya
Source: Int J Parasitol Parasites Wildl. 2026 Jan 14;29:101191. doi: 10.1016/j.ijppaw.2026.101191 (PMC12856993; doi:10.1016/j.ijppaw.2026.101191)
Supplement: Multimedia component 1 [file mmc1.pdf]

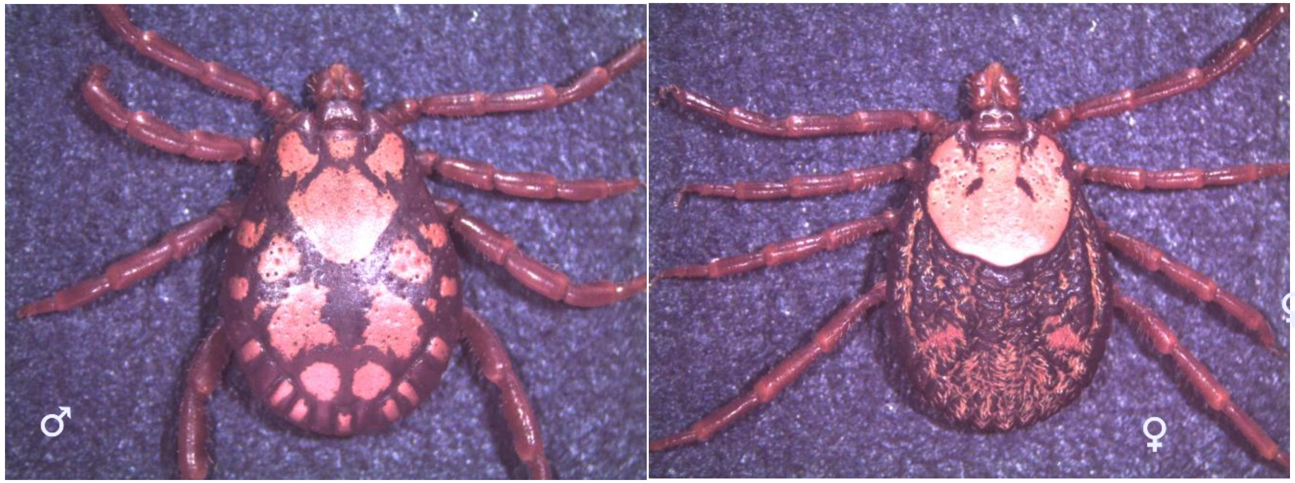

Dermacentor rhinocerinus

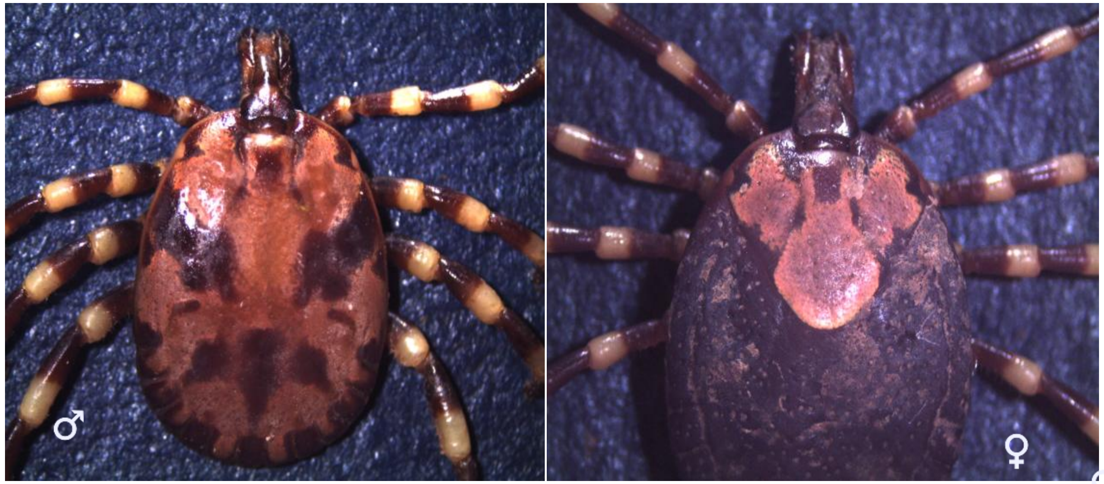

Amblyomma rhinocerotis

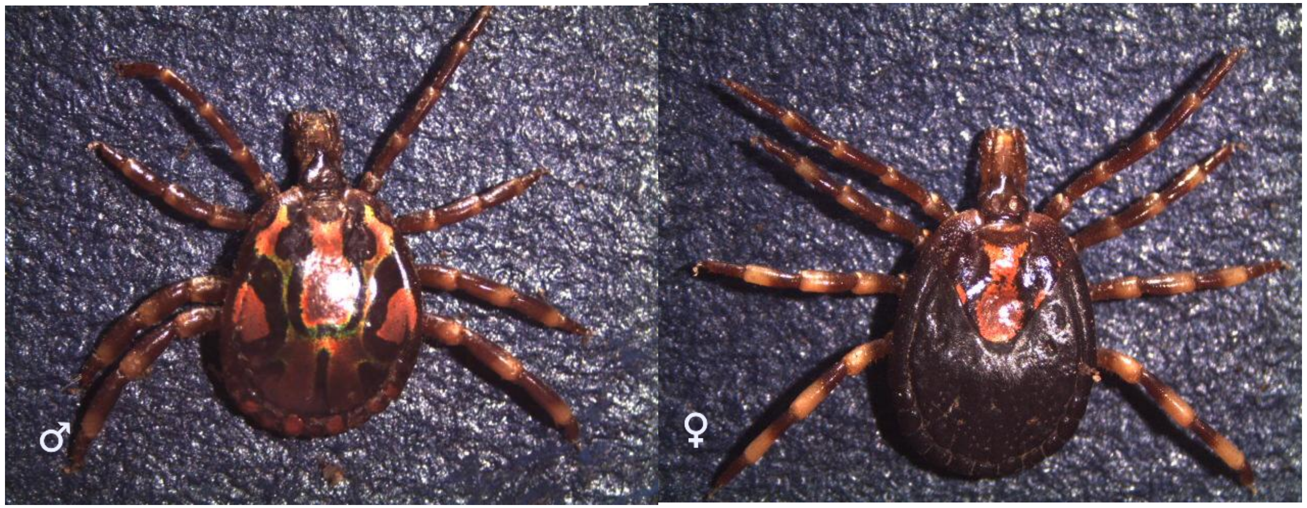

Amblyomma cohaerens

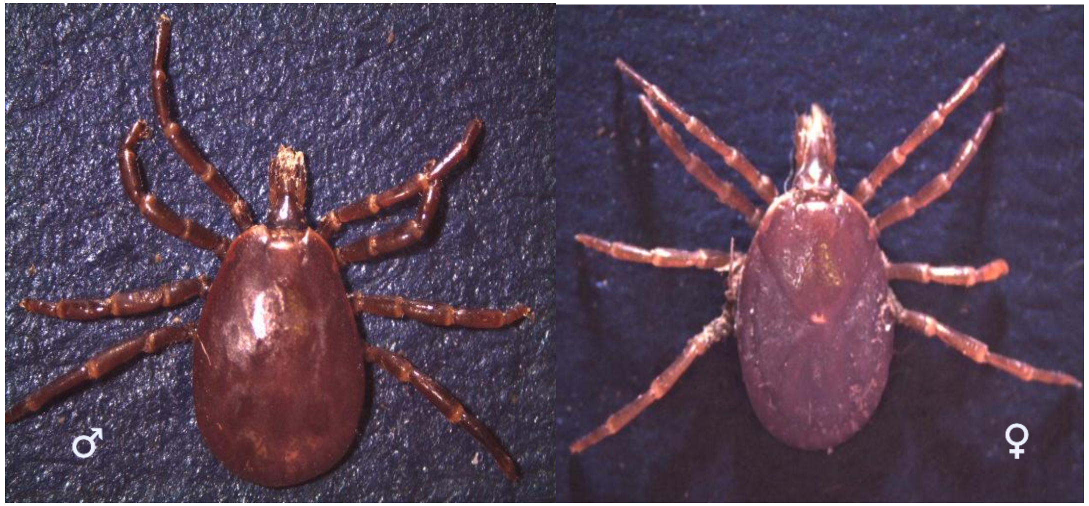

Amblyomma tholloni

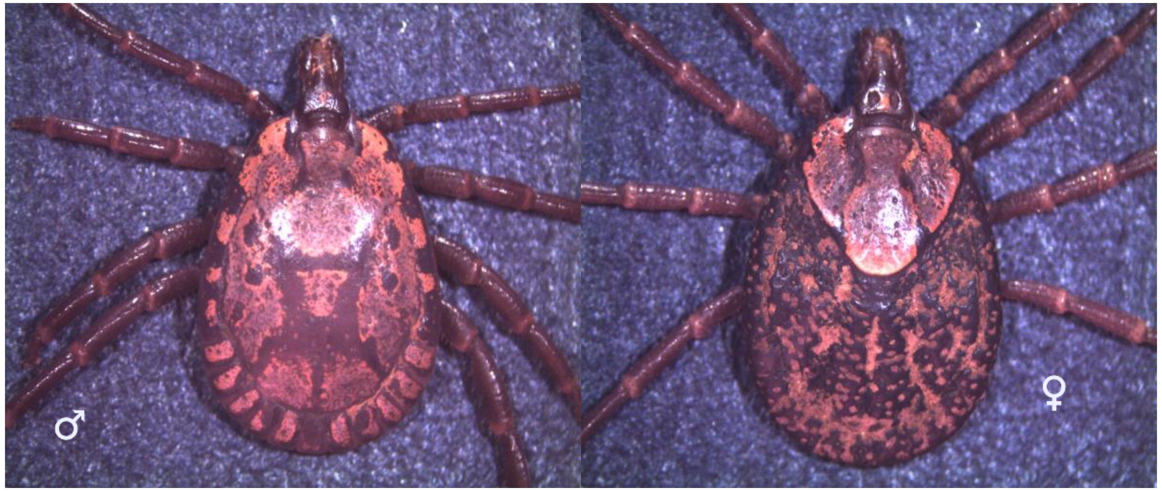

Amblyomma sparsum

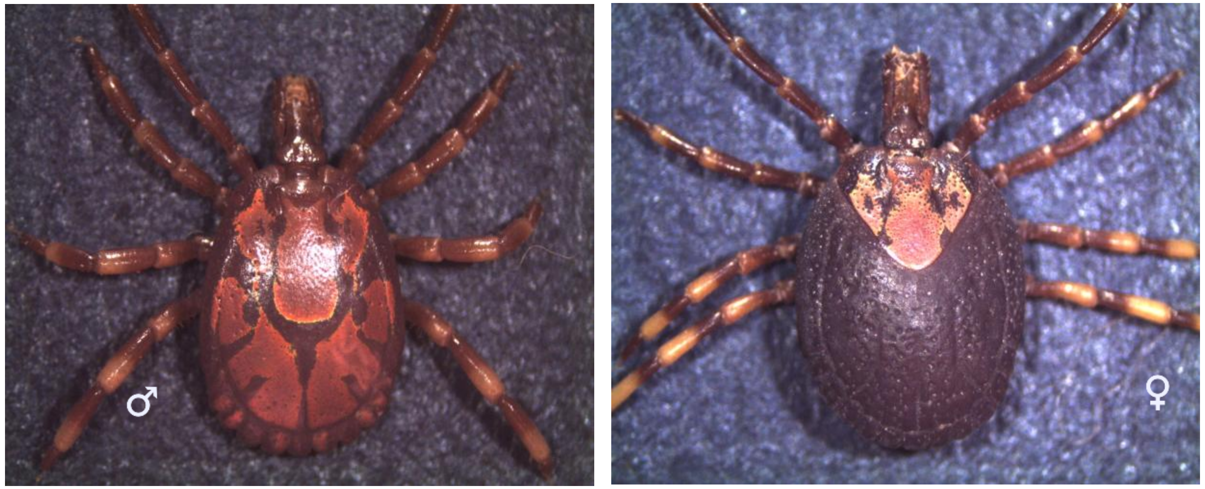

Amblyomma gemma

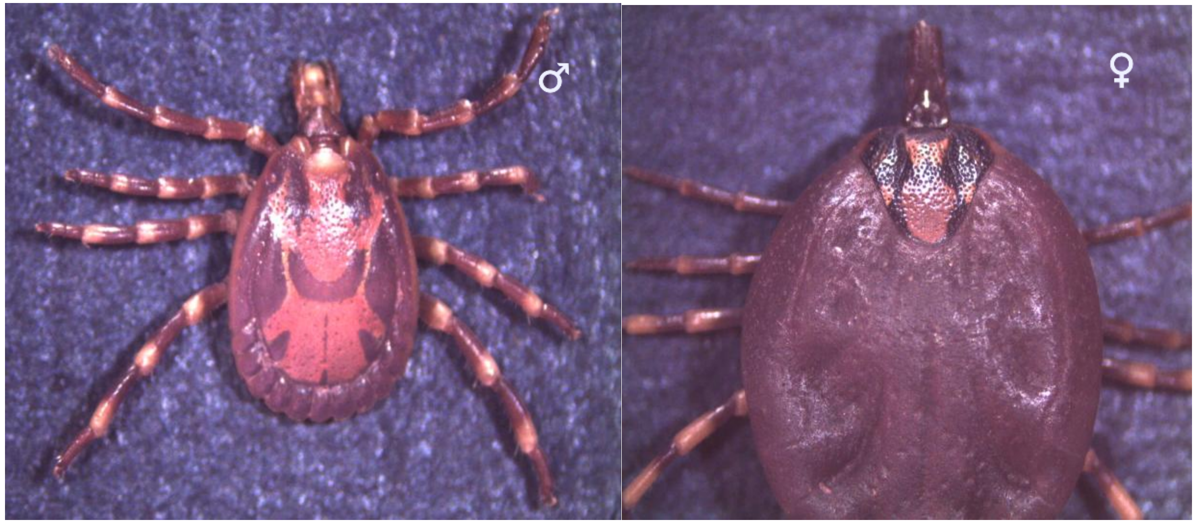

Amblyomma variegatum

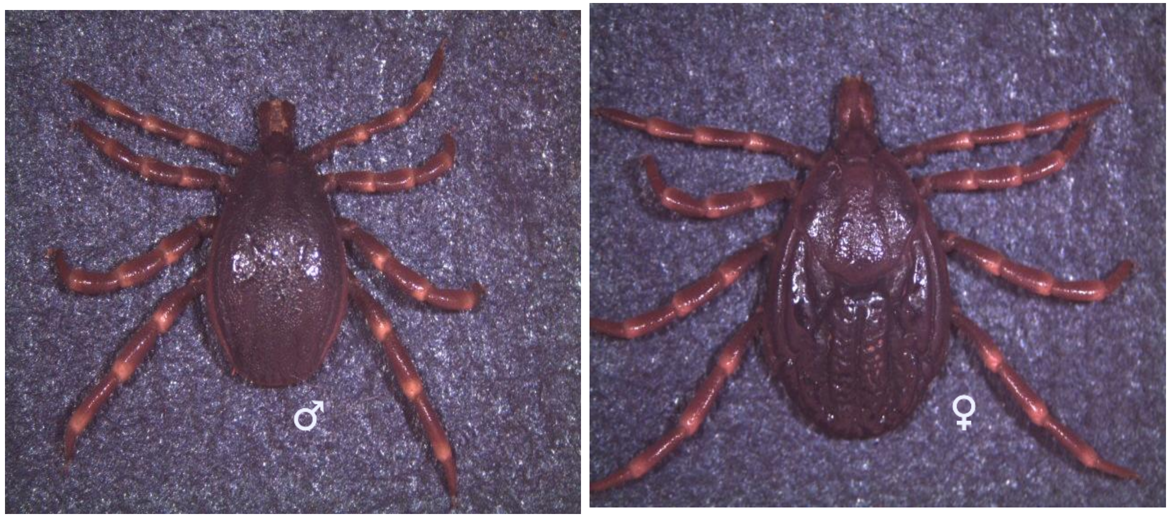

Hyalomma rufipes

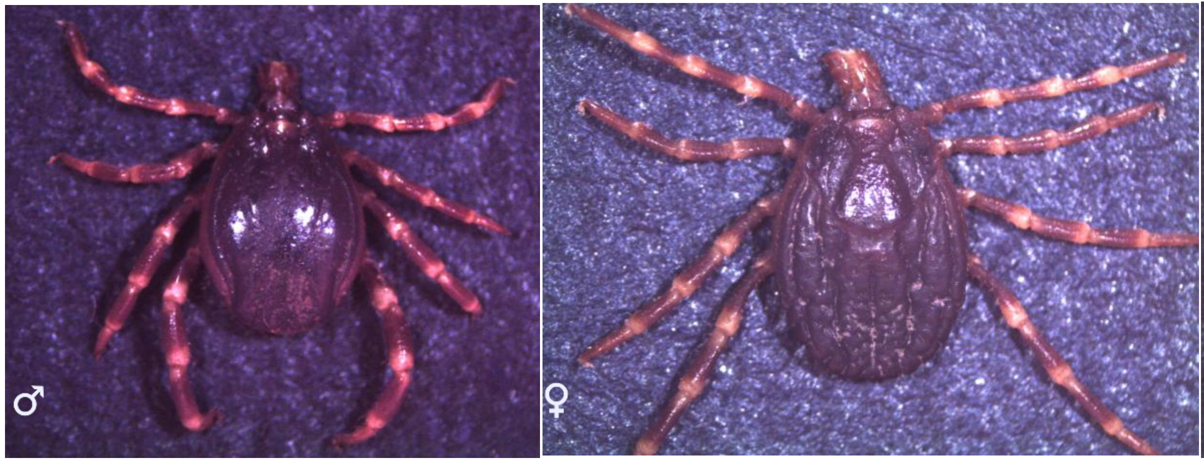

Hyalomma truncatum

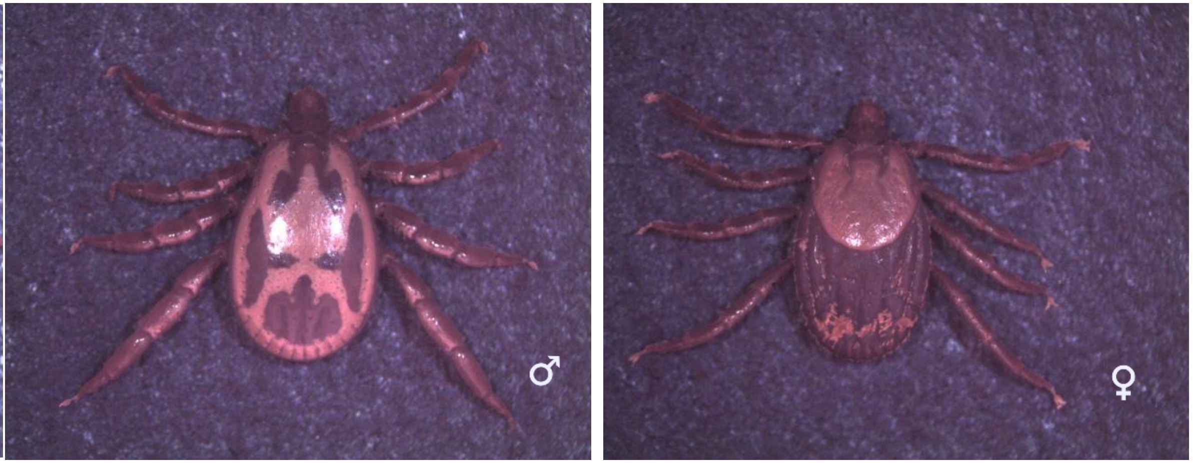

Rhipicephalus pulchellus

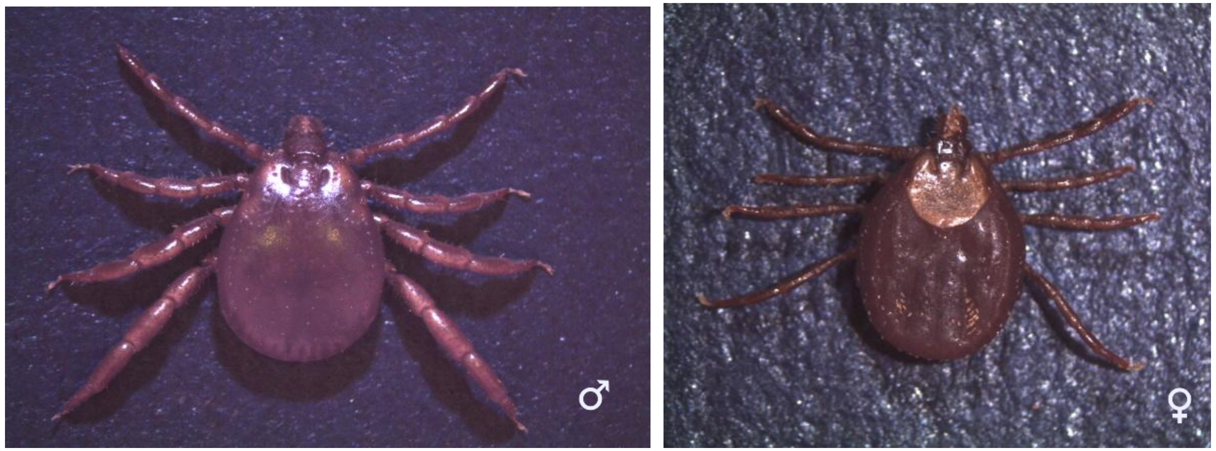

Rhipicephalus humeralis

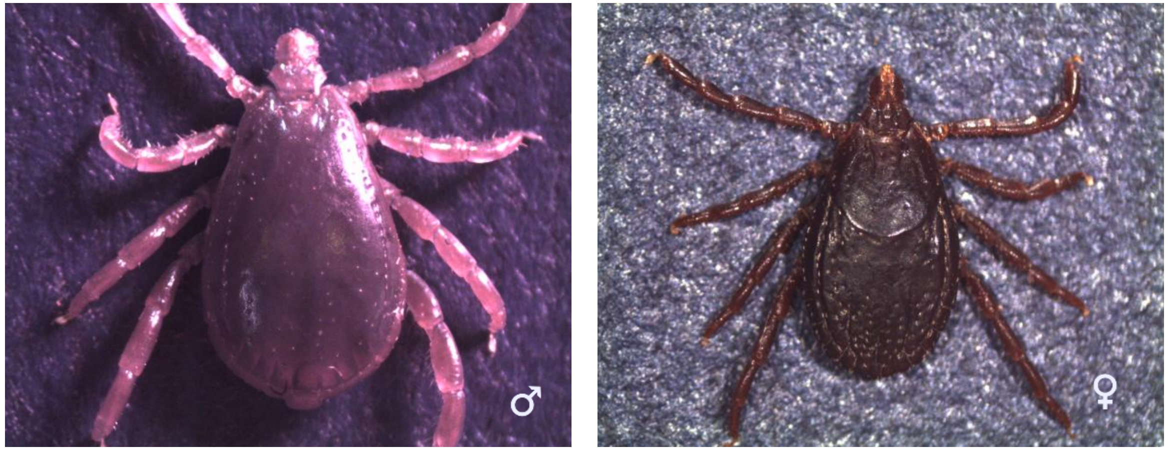

Rhipicephalus praetextatus

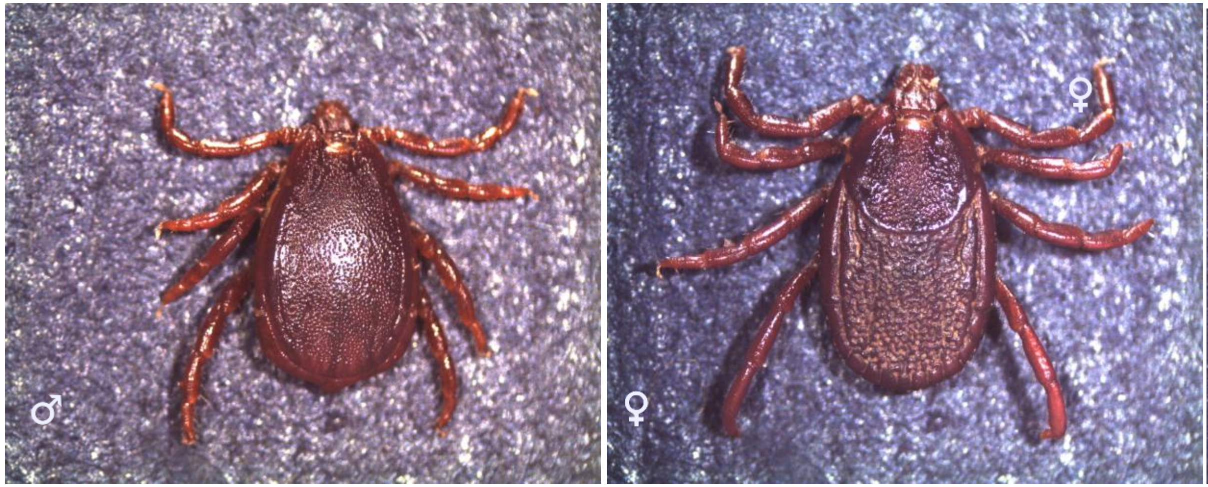

Rhipicephalus compositus

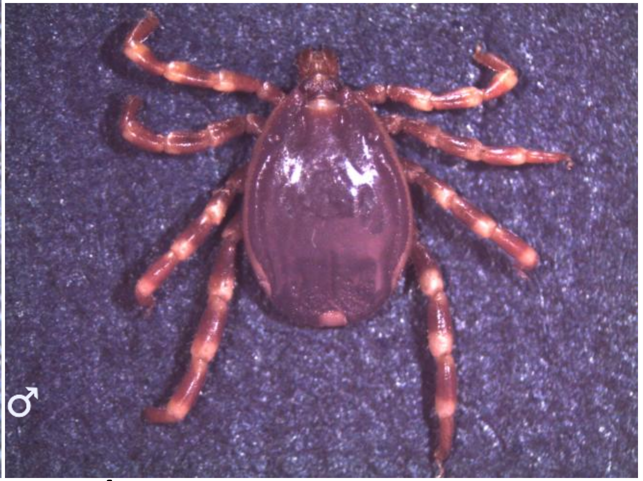

Hyalomma albiparmatum

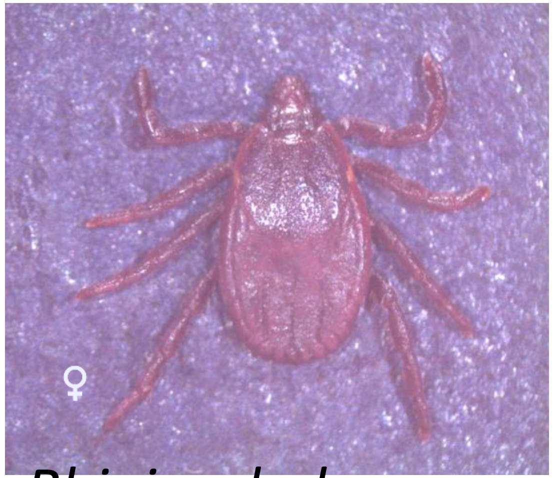

Rhipicephalus appendiculatus

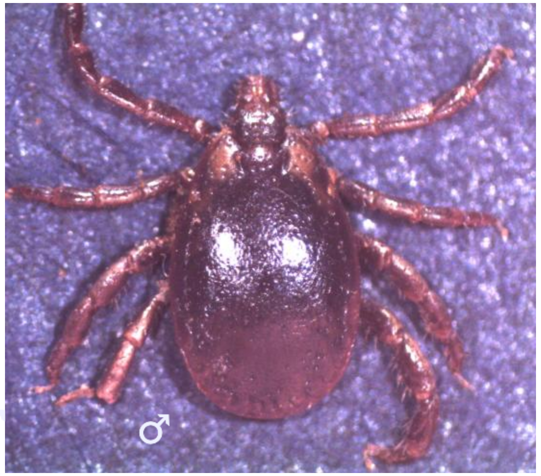

Rhipicephalus jeanneli

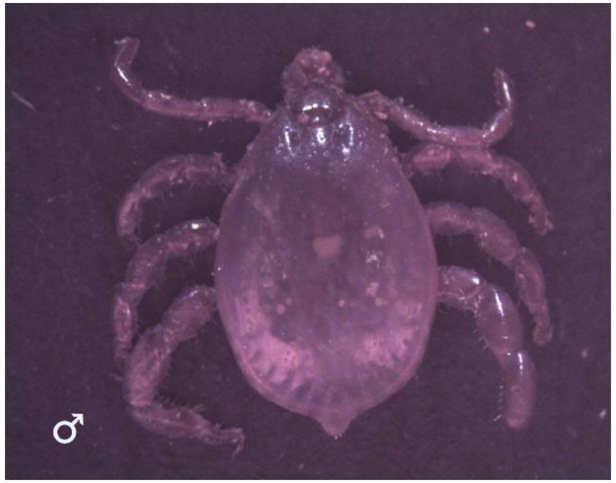

Rhipicephalus maculatus

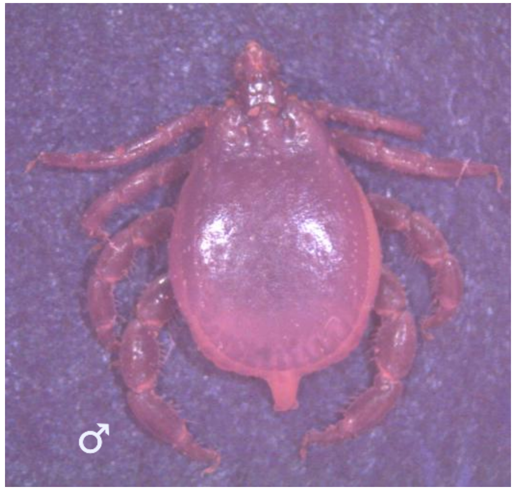

Rhipicephalus muehlensi

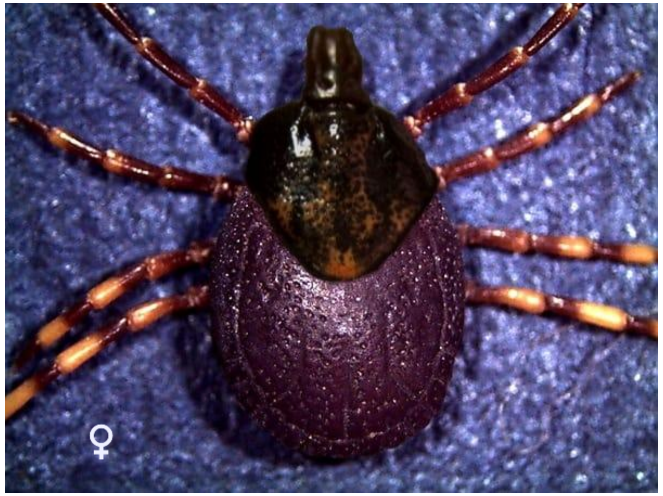

Amblyomma lepidum

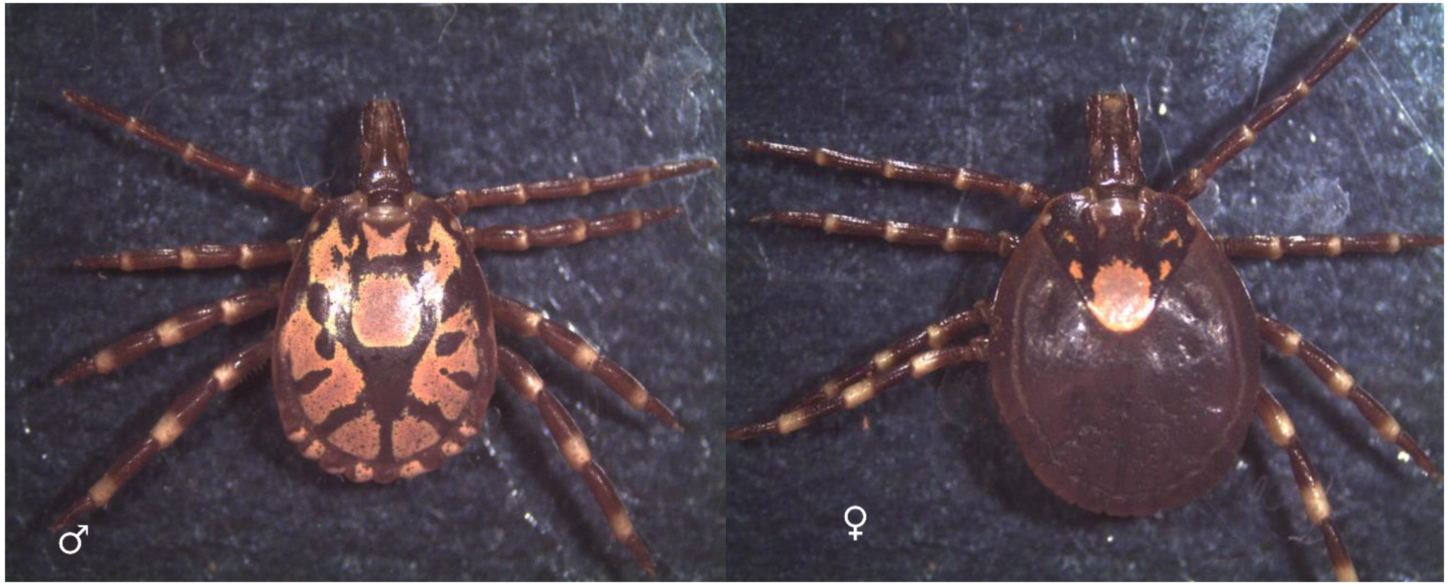

Amblyomma eburneum
