# Supplementary material for: Drivers of tick community structure in a rhinoceros meta-population in Kenya
Source: Int J Parasitol Parasites Wildl. 2026 Jan 14;29:101191. doi: 10.1016/j.ijppaw.2026.101191 (PMC12856993; doi:10.1016/j.ijppaw.2026.101191)
Supplement: Multimedia component 2 [file mmc2.docx]

**Table S1**: Tick sampling from rhinoceros showing the numbers sampled, rhinoceros management activities and the species of rhinoceros involved.

| **Sanctuary** | **Month-Year** | **Reason for Immobilization** |  | **Black rhinoceros** | **White rhinoceros** | **Rhinoceros sampled** |
| --- | --- | --- | --- | --- | --- | --- |
| IPZ | Jul - 2022 | Ear notching | 11 | NA | 11 |  |
|  | Nov - 2024 |  | 14 | NA | 14 | 25 |
| LBL | Mar - 2023 | Ear notching | 19 | 23 | 42 |  |
|  | May - 2025 |  | 15 | 23 | 38 |  |
|  | Jan - 2024 | Translocation | 11 | 0 | 11 |  |
|  | Feb - 2024 |  | 0 | 4 | 4 |  |
|  | May - 2025 |  | 5 | 0 | 5 |  |
|  | Jun - 2025 |  | 4 | 0 | 4 |  |
|  | Aug - 2025 |  | 0 | 6 | 6 |  |
|  | Oct - 2025 |  | 0 | 2 | 2 | 112 |
| LNP | Jan - 2018 | Ear notching | 8 | 0 | 8 |  |
|  | Oct - 2021 |  | 8 | 3 | 11 |  |
|  | Jun - 2025 | Translocation | 3 | 0 | 3 |  |
|  | Feb - 2024 | Treatment | 1 | 0 | 1 | 23 |
| MNP | Aug - 2021 | Ear notching | 4 | 14 | 18 |  |
|  | Aug - 2025 | Translocation | 0 | 4 | 4 |  |
|  | Mar - 2025 | Treatment | 0 | 1 | 1 | 23 |
| MNR | Feb - 2016 | Ear notching | 3 | NA | 3 |  |
|  | Feb - 2022 |  | 1 | NA | 1 |  |
|  | Feb - 2025 |  | 13 | NA | 13 |  |
|  | Mar - 2024 |  | 7 | NA | 7 |  |
|  | Oct - 2021 |  | 2 | NA | 2 | 26 |
| NNP | Jun - 2021 | Ear notching | 1 | 0 | 1 |  |
|  | Jul - 2021 |  | 16 | 7 | 23 |  |
|  | Feb - 2024 | Translocation | 3 | 0 | 3 |  |
|  | Mar - 2025 |  | 1 | 0 | 1 |  |
|  | Aug - 2020 |  | 1 | 0 | 1 |  |
|  | Feb - 2024 | Treatment | 0 | 1 | 1 | 30 |
| NRS | Jul - 2022 | Ear notching | 3 | NA | 3 |  |
|  | Nov - 2024 |  | 14 | NA | 14 | 17 |
| OLJ | Jun - 2022 | Ear notching | 16 | 10 | 26 |  |
|  | Oct - 2022 | Translocation | 0 | 12 | 12 | 38 |
| OPC | Feb - 2023 | Ear notching | 26 | 10 | 36 |  |
|  | Mar - 2023 |  | 0 | 2 | 2 |  |
|  | May - 2023 |  | 0 | 1 | 1 |  |
|  | Jan - 2024 | Translocation | 7 | 0 | 7 |  |
|  | May - 2025 |  | 9 | 0 | 9 | 55 |
| SER | Jul - 2023 | Ear notching | 6 |  | 6 | 6 |
| SRS | Oct - 2025 | De-snaring |  | 1 | 1 |  |
|  | May - 2025 | Translocation |  | 1 | 1 |  |
|  | Jun - 2025 | Translocation | 1 |  | 1 |  |
|  | Sep - 2025 | Translocation |  | 7 | 7 |  |
|  | Nov - 2025 | Translocation |  | 1 | 1 | 11 |
| TEN | Jan - 2021 | Ear notching | 6 |  | 6 | 6 |
| All |  | Various | 239 | 133 | 372 | 372 |
