# Supplementary material for: Drivers of tick community structure in a rhinoceros meta-population in Kenya
Source: Int J Parasitol Parasites Wildl. 2026 Jan 14;29:101191. doi: 10.1016/j.ijppaw.2026.101191 (PMC12856993; doi:10.1016/j.ijppaw.2026.101191)
Supplement: Multimedia component 3 [file mmc3.docx]

**Table S2.** Statistically significant variations in species richness of ticks infesting rhinoceros among Sanctuaries in Kenya

|  | IPZ | LBL | LNP | MNP | MNR | NNP | NRS | OLJ | OPC | SER | SRS |
| --- | --- | --- | --- | --- | --- | --- | --- | --- | --- | --- | --- |
| IPZ |  |  |  |  |  |  |  |  |  |  |  |
| LBL | 0.119 |  |  |  |  |  |  |  |  |  |  |
| LNP | **0.000** | **0.006** |  |  |  |  |  |  |  |  |  |
| MNP | **0.008** | 0.721 | 0.960 |  |  |  |  |  |  |  |  |
| MNR | **0.016** | 0.973 | 0.668 | 1.000 |  |  |  |  |  |  |  |
| NNP | 1.000 | 0.555 | **0.000** | **0.043** | 0.132 |  |  |  |  |  |  |
| NRS | 1.000 | 0.051 | **0.000** | **0.002** | **0.009** | 0.996 |  |  |  |  |  |
| OLJ | **0.000** | **0.011** | 1.000 | 1.000 | 0.982 | **0.000** | **0.000** |  |  |  |  |
| OPC | **0.000** | 0.542 | 0.553 | 1.000 | 1.000 | **0.012** | **0.000** | 0.963 |  |  |  |
| SER | **0.000** | **0.016** | 0.998 | 0.550 | 0.299 | **0.000** | **0.000** | 0.866 | 0.282 |  |  |
| SRS | 0.623 | 1.000 | 0.574 | 1.000 | 1.000 | 0.944 | 0.358 | 0.918 | 1.000 | 0.226 |  |
| TEN | 1.000 | 1.000 | 0.158 | 0.836 | 0.954 | 1.000 | 1.000 | 0.379 | 0.886 | 0.054 | 1.000 |
