# Supplementary material for: Drivers of tick community structure in a rhinoceros meta-population in Kenya
Source: Int J Parasitol Parasites Wildl. 2026 Jan 14;29:101191. doi: 10.1016/j.ijppaw.2026.101191 (PMC12856993; doi:10.1016/j.ijppaw.2026.101191)
Supplement: Multimedia component 4 [file mmc4.docx]

**Table S3.** Statistically significant variations in asymptotic Hill-Shannon diversity (upper diagonal) and Hill-Simpson

diversity (lower diagonal) of ticks infesting rhinoceros in Kenya’s rhinoceros sanctuaries

|  | IPZ | LBL | LNP | MNP | MNR | NNP | NRS | OLJ | OPC | SER | SRS | TEN |
| --- | --- | --- | --- | --- | --- | --- | --- | --- | --- | --- | --- | --- |
| IPZ |  | 0.181 | **0.000** | **0.000** | **0.000** | 1.000 | 0.607 | **0.003** | 0.054 | **0.002** | 0.984 | 1.000 |
| LBL | 0.576 |  | 0.140 | 0.133 | **0.011** | 0.286 | **0.000** | 0.502 | 1.000 | 0.120 | 1.000 | 0.998 |
| LNP | **0.016** | 0.446 |  | 1.000 | 1.000 | **0.000** | **0.000** | 1.000 | 0.735 | 0.997 | 0.668 | 0.308 |
| MNP | **0.001** | 0.085 | 1.000 |  | 1.000 | **0.000** | **0.000** | 1.000 | 0.718 | 0.997 | 0.662 | 0.299 |
| MNR | **0.000** | **0.006** | 1.000 | 1.000 |  | **0.000** | **0.000** | 0.980 | 0.244 | 1.000 | 0.344 | 0.145 |
| NNP | 1.000 | 0.742 | **0.028** | **0.006** | **0.000** |  | 0.322 | **0.007** | 0.094 | **0.006** | 0.998 | 1.000 |
| NRS | 0.239 | **0.000** | **0.000** | **0.000** | **0.000** | 0.111 |  | **0.000** | **0.000** | **0.000** | 0.083 | 0.926 |
| OLJ | 0.101 | 0.954 | 1.000 | 0.959 | 0.496 | 0.140 | **0.000** |  | 0.997 | 0.833 | 0.960 | 0.641 |
| OPC | 0.424 | 1.000 | 0.861 | 0.348 | **0.043** | 0.581 | **0.000** | 1.000 |  | 0.332 | 1.000 | 0.958 |
| SER | **0.027** | 0.292 | 1.000 | 1.000 | 1.000 | **0.041** | **0.000** | 0.879 | 0.485 |  | 0.250 | 0.105 |
| SRS | 1.000 | 1.000 | 0.878 | 0.539 | 0.200 | 1.000 | 0.076 | 0.998 | 1.000 | 0.453 |  | 1.000 |
| TEN | 1.000 | 1.000 | 0.678 | 0.388 | 0.166 | 1.000 | 0.784 | 0.948 | 1.000 | 0.292 | 1.000 |  |
